# Supplementary material for: Coping and support-seeking in out-of-home care: a qualitative study of the views of young people in care in England
Source: BMJ Open. 2021 Feb 15;11(2):e038461. doi: 10.1136/bmjopen-2020-038461 (PMC7887338; doi:10.1136/bmjopen-2020-038461)
Supplement: Supplementary data [file bmjopen-2020-038461supp001.pdf]

Child qualitative interview for C-CATS - version 1

Child qualitative interview

Thank you for talking to me today. Like it said in the letter I'm interested in finding out a bit more about your views on how you cope and where you might go for support or help if you're struggling with thoughts or feelings. Remember, you don't need to talk about your experiences in your home. You can if you want to but we won't be asking you to. What I'm really interested in is what happened *after* this, when you came in to care. This isn't like a test at school, there are no right or wrong answers, it's just about talking honestly and sharing your opinion. If you want to stop at any time or take a break just let me know. Does that sound ok?

Demographics

- Do you remember how old you were when you first came in to care?
- How long have you lived in [current placement]?

Reactions

- How have you felt since you came in to care?
    - Are there good parts?
    - What are some of the harder parts?

Support

- If you're having a hard time, is there anything you do to try and make yourself feel better or different?
    - (*example might be argument with friend at school; through to mental health difficulty if yp has disclosed this*)

- Is there anyone you talk to, to try to make yourself feel better?
    - Who? Is that helpful/unhelpful? Why?
    - Anyone else?
    - Have you ever talked to a professional? Like a psychologist or school counsellor? What was this like? What was helpful/unhelpful?

- Do you ever talk to anyone about what you experienced before you came into care?
    - Why/why not?
    - What helps/doesn't help?

Support wishes

- What support do you wish you had got that you didn't?
    - (example might be – *if you were asked to create the best possible care system to support young people who were struggling with their thoughts and feelings, what would it look like?*)
- If you had a friend that went through the same things as you, how would you help them? What advice might you give them to help them cope with their thoughts and feelings?

Is there anything else you want to add that we haven't covered?

Hiller RM, *et al. BMJ Open* 2021; 11:e038461. doi: 10.1136/bmjopen-2020-038461
