# Supplementary material for: Coping and support-seeking in out-of-home care: a qualitative study of the views of young people in care in England
Source: BMJ Open. 2021 Feb 15;11(2):e038461. doi: 10.1136/bmjopen-2020-038461 (PMC7887338; doi:10.1136/bmjopen-2020-038461)
Supplement: Author's manuscript [file bmjopen-2020-038461.draft_revisions.pdf]

BMJ Open is committed to open peer review. As part of this commitment we make the peer review history of every article we publish publicly available.

When an article is published we post the peer reviewers' comments and the authors' responses online. We also post the versions of the paper that were used during peer review. These are the versions that the peer review comments apply to.

The versions of the paper that follow are the versions that were submitted during the peer review process. They are not the versions of record or the final published versions. They should not be cited or distributed as the published version of this manuscript.

BMJ Open is an open access journal and the full, final, typeset and author-corrected version of record of the manuscript is available on our site with no access controls, subscription charges or pay-per-view fees (http://bmjopen.bmj.com).

If you have any questions on BMJ Open's open peer review process please email info.bmjopen@bmj.com

BMJ Open

Coping and support-seeking in out-of-home care: A qualitative study of the views of young people in care in England

Journal:	*BMJ Open*
Manuscript ID	bmjopen-2020-038461
Article Type:	Original research
Date Submitted by the Author:	13-Mar-2020
Complete List of Authors:	Hiller, Rachel; University of Bath, Department of Psychology<br>Halligan, Sarah; University of Bath, Psychology; University of Cape Town, Department of Psychiatry and Mental Health<br>Meiser-Stedman , Richard ; University of East Anglia,<br>Elliott, Elizabeth; University of Bath, Department of Psychology; University of Reading, School of Psychology and Clinical Language Science<br>Rutter-Eley, Emily; University of Reading, School of Psychology and Clinical Language Science<br>Hutt, Tilly; University of Bath, Department of Psychology
Keywords:	Child protection < PAEDIATRICS, Child & adolescent psychiatry < PSYCHIATRY, Quality in health care < HEALTH SERVICES ADMINISTRATION & MANAGEMENT

SCHOLARONE™
Manuscripts

I, the Submitting Author has the right to grant and does grant on behalf of all authors of the Work (as defined in the below author licence), an exclusive licence and/or a non-exclusive licence for contributions from authors who are: i) UK Crown employees; ii) where BMJ has agreed a CC-BY licence shall apply, and/or iii) in accordance with the terms applicable for US Federal Government officers or employees acting as part of their official duties; on a worldwide, perpetual, irrevocable, royalty-free basis to BMJ Publishing Group Ltd ("BMJ") its licensees and where the relevant Journal is co-owned by BMJ to the co-owners of the Journal, to publish the Work in this journal and any other BMJ products and to exploit all rights, as set out in our licence.

The Submitting Author accepts and understands that any supply made under these terms is made by BMJ to the Submitting Author unless you are acting as an employee on behalf of your employer or a postgraduate student of an affiliated institution which is paying any applicable article publishing charge ("APC") for Open Access articles. Where the Submitting Author wishes to make the Work available on an Open Access basis (and intends to pay the relevant APC), the terms of reuse of such Open Access shall be governed by a Creative Commons licence – details of these licences and which Creative Commons licence will apply to this Work are set out in our licence referred to above.

Other than as permitted in any relevant BMJ Author's Self Archiving Policies, I confirm this Work has not been accepted for publication elsewhere, is not being considered for publication elsewhere and does not duplicate material already published. I confirm all authors consent to publication of this Work and authorise the granting of this licence.

For peer review only

Coping and support-seeking in out-of-home care: A qualitative study of the views of young people in care in England

Rachel M Hiller[1], Sarah L Halligan[1,2], Richard Meiser-Stedman[3], Elizabeth Elliott[1,4], Emily Rutter-Eley[4], Tilly Hutt[1]

[1] Department of Psychology, University of Bath, UK

[2] Department of Psychiatry and Mental Health, University of Cape Town, South Africa

[3] Department of Clinical Psychology, Norwich Medical School, University of East Anglia, UK

[4] School of Psychology and Clinical Language Science, University of Reading, UK

* Corresponding Author:

Dr Rachel Hiller

Department of Psychology

University of Bath

P. 01225 383794

E. R.Hiller@bath.ac.uk

Main text word count: 5, 101

For peer review only - http://bmjopen.bmj.com/site/about/guidelines.xhtml

Abstract

Objectives: Young people who have been removed from their family home and placed in out-of-home care have often experienced maltreatment. High rates of mental health difficulties have been well-documented in this group. The main aim of this research was to explore the experiences of these young people within the care system, particularly in relation to support-seeking and coping with emotional needs, to better understand feasible and acceptable ways to improve outcomes for this group.

Design and Study-Setting: This study used 1:1 semi-structured qualitative interviews with young people in out-of-home care in England, to provide an in-depth understanding of their views of coping and support for their emotional needs, both in terms of support networks and experiences with mental health services. Participants were 25 10-16 years olds (56% female) and included young people living with non-biological foster carers, kinship carers, and in residential group homes.

Results: Participants described positive (e.g. feeling safe) and negative (e.g. feeling judged) aspects to being in care. Carers were identified as the primary source of support, with a supportive adult central to coping. Views on support and coping differed for young people who were experiencing more significant mental health difficulties, with this group largely reporting feeling unsupported and many engaging in self-harm. The minority of participants had accessed formal mental health support, and opinions on usefulness were mixed.

Conclusions: Results are discussed in terms of practical implications and avenues for improving the wellbeing of this group.

Key words: child maltreatment; Looked after Children; foster care; emotional wellbeing; mental health; qualitative

For peer review only - http://bmjopen.bmj.com/site/about/guidelines.xhtml

Strengths and Limitations of the Study:

- This study is focused on young people living in out-of-home care, represented an under-researched group where there remains significant need.
- We used qualitative interview methods to gain in-depth understanding of the views and experiences of these young people.
- As this study uses qualitative methods, we cannot conclude whether different types of reported coping might be associated with better or worse outcomes.
- The study recruited from two English Local Authorities.

For peer review only

For peer review only - http://bmjopen.bmj.com/site/about/guidelines.xhtml

Introduction

The most common reason a child would be removed from their home and placed in out-of-home care is due to the experience of abuse and/or neglect (Department for Education, 2017; Oswald, Heil & Goldbeck, 2009; Pecora et al., 2009). In the UK, it is also the case that most young people who enter care are removed from them home when school-aged or older, meaning early experiences of maltreatment have often been ongoing for a number of years (Department for Education, 2017). Once in care many continue to face ongoing instability. Separation from siblings is common, as are moves between different placement providers, resulting in the potential for inconsistent support (Hiller & St Clair, 2018; Newton, Litrownik & Landsverk, 2000). Given these accumulative experiences, it is perhaps unsurprising that there is well-established evidence that young people in out-of-home care experience high rates of psychological difficulties compared to their peers (e.g., Ford et al., 2007; McMillen et al., 2005; Oswold et al., 2009).

Failing to adequately address the psychology or emotional needs of young people in care has been identified as a key driver of many of the well-documented poor outcomes that can categorise this group, including school attainment and later rates of homelessness and unemployment (Jones et al., 2011). Yet, despite the long-documented poor outcomes, there has been limited evidence of improvements in the overall picture of mental health for children in care in the UK over the past decade (Department for Education, 2017). One concern is that these young people can fall between the gaps of social-care and national health service provisions, which can result in poor access to mental health services (Davies & Wright, 2008; Shaw & DeJong, 2012). With resource issues affecting child and adolescent mental health services (CAMHS) in many countries, it is also largely impossible for these services to address the large number of young people in care who may benefit from professional mental health support.

For peer review only - http://bmjopen.bmj.com/site/about/guidelines.xhtml

In the face of potentially limited professional support services, young people will inevitably develop day-to-day coping strategies and support networks – whether perceived to be adaptive or maladaptive. Some qualitative work has investigated the views of young people in care in relation to support networks, and specifically their relationships with carers, finding developing trust, consistent boundaries, and empathy were viewed as important (e.g., Ahmed, Windsor & Scott, 2015; Sinclair & Wilson, 2003). However, there is less evidence for how they might engage with other support networks, including peers, school, and social workers. Such evidence is potentially particularly important for this group, given one-third of young people in care in the UK will have more than one carer in any given year (Department for Education, 2017), and given the well-documented cyclical association between placement breakdowns and emotional difficulties (Newton, Litrownik & Landsverk, 2000; Rock et al., 2013). Beyond the role of external support networks, we know little about what strategies young people in care may be using themselves to manage their emotional wellbeing. Understanding how young people in care cope with emotional difficulties, particularly in the face of potentially disrupted support networks, is crucial to understanding the experiences of this group and avenues for improving formal and informal support, particularly given evidence from the wider child mental health field that certain types of coping, especially avoidance, can exacerbate mental health difficulties (e.g., Murray et al., 2009; Trickey et al., 2012), including in care-experienced youth (Hiller et al., 2020).

We used a semi-structured qualitative approach to explore in-depth the experiences of young people in care. While this included a general exploration of what young people viewed as the pros and cons of being in care, the research questions were primarily focused on: (1) where do young people seek support for emotional difficulties, both in terms of social support and professional services?; (2) what do they view as barriers to seeking help?; and (3) what coping strategies do they use when experiencing emotional difficulties? Discussion on coping

For peer review only - http://bmjopen.bmj.com/site/about/guidelines.xhtml

with emotional difficulties included both more common day-to-day experiences (e.g., if they feel upset or stressed) and more significant and prolonged mental health difficulties.

Method

Recruitment and Sample

Ethical approval was granted from the University of Bath and Social Care research ethics committees, along with permission from the participating Local Authorities. Using opportunity sampling, we recruited 25 young people aged 10 – 16 years old. Participants were all part of a larger quantitative study on the mental health and wellbeing of young people in care (Hiller et al., 2020). The sample size is based on general guidance for qualitative methodology (Vasileiou et al., 2018). Participants came from two Local Authorities in England. One was a smaller-sized Local Authority and one was medium-sized, with both in largely Urban areas. Informed consent for the young person's participation was provided by their social worker, while the young person provided informed assent (or consent if 16 years old). Eligibility for participation in the quantitative study were: aged 10-17 years old, on full care order (unless 16+ years old), absence of developmental disability that would preclude the child from mainstream schooling, and absence of significant current risk (e.g., current severe suicidality). Young people could be in any style of placement, including foster placement with a non-biological carer, kinship placement, residential care home. Descriptive information on the sample is presented in Table 1.

Procedure and Measures

In-person 1:1 interviews were conducted by two researchers. The lead researcher (RH) had prior training and experience in qualitative research, while the other (EE) was experienced researching with vulnerable young people and adults and received further training specifically on qualitative procedures. We used a 1:1 semi-structured interview format, rather than focus group, due to the potential for sensitive information being discussed

For peer review only - http://bmjopen.bmj.com/site/about/guidelines.xhtml

that young people may not wish to disclose in a group. For one of these interviews two siblings requested to complete the interview together, while for two interviews the carer remained present at the request of the young person. All others (n = 22) had only the young person and interviewer present. RH and EE met regularly throughout the study to monitor the quality of the interviews and reflect on the content. All interviews were audio recorded and later transcribed verbatim. As a thank you for volunteering their time, the young people each received a £10 voucher. As per standard risk management and safeguarding procedures, where appropriate, summary letters were provided to social workers to support potential referrals to mental health services.

Descriptives and semi-structured interview. Basic descriptive information was collected from the young person's social worker, as part of the larger quantitative study. This included the age and reason that they entered care. The semi-structured interview was developed based on the literature on qualitative interviewing techniques and qualitative and quantitative work in the child mental health field (e.g., Davies & Wright, 2008; Docherty & Sandelowski, 1999; Van Wesel et al., 2012). Interviews broadly covered three core areas: (i) views on what is broadly helpful and unhelpful within the care system, (ii) coping and support for children's mental health and wellbeing (including at home, within social-care, and experiences around mental health services), (iii) coping and support for trauma-related mental health more specifically (e.g., how young people cope with memories of their maltreatment), and (iv) views on ideal support. Questions were primarily open-ended (e.g., "If you're having a hard time what do you do to make yourself feel better or different?"). Some direct prompting was used to clarify comments (e.g., "Was that helpful?"). Interviews lasted from 6-30 minutes.

Data Analyses

For peer review only - http://bmjopen.bmj.com/site/about/guidelines.xhtml

Transcripts were analysed using thematic analysis (Braun & Clarke, 2006) in NVivo 12. Co-author (ER), who was not involved in any of the interviews, coded all transcripts. She first read all transcripts to gain an understanding of the overall data (data immersion) and then systematically coded each transcript. Given the exploratory nature of the study, thematic analysis was chosen as the data analytic approach as it allows for a detailed exploration of the data and does not rely on a specific theoretical framework. Following the systematic coding of all transcripts, codes were grouped to form overarching themes. Co-authors [initials removed] then independently explored all (un-coded) transcripts and generated their own key themes. A consensus meeting showed strong agreement between the primary themes. There was not capacity within the study to seek further input from participants at this point, however reflective practices were used throughout all interviews to ensure clear understanding of the discussion and to minimise any chance of misinterpreting what was being said (e.g., reflecting, summarising back to participants and seeking further clarification where needed).

Patient and Public Involvement

This study occurred as part of a series of projects, which involves extensive consultation with services, as well as feedback from care-experienced young people and foster carers. This feedback particularly shaped the research questions and use of qualitative methods. Young people and carers were not involved in recruitment for the project. There has been wide written and verbal feedback to stake-holders as part of the larger project.

Results

Descriptive Information

The sample comprised of 14 females and 11 males, aged 10 to 16 years old. They had entered care between infancy and 16 years old, and had been in care from 6 months to almost 14 years, while 80% of the sample had had more than one placement since entering care.

For peer review only - http://bmjopen.bmj.com/site/about/guidelines.xhtml

Most (76%) were living in a foster placement with a non-biological carer, while four were in a kinship placement (e.g., with a grandmother), and two lived in residential care homes. All were in care due to the experience of abuse and/or neglect.

Interview Themes

Thematic analysis identified three primary themes covering: being in care had both positive and negative aspects (Theme 1); social support was key to coping, but not always available (Theme 2); and that adaptive and maladaptive coping strategies were used to manage emotional difficulties (Theme 3). A list of themes and subthemes can be found in Table 2.

Theme 1: Being in care has positive and negative aspects

When asked what it was like to be in care, most, but not all participants expressed that being in care was a better or safer alternative to living with their biological family and that they felt safer in care. However, almost all discussed occasions of feeling stigmatised, as well as experiences of being poorly supported by services.

Subtheme 1: Being in care can create a sense of safety and of being part of a family. The majority of participants reported that feeling safe and secure was a particularly positive aspect of being in care, relating to the general acceptance from most participants that being in care was the safest option for them at this current time. Some participants also described experiencing a sense of relief when first coming into care, recognising that it was not appropriate for them to remain in an unsafe environment.

Relieved actually from going to a bad environment into somewhere I thought could be quite safe.

To know at the end of the day you can go home, instead of thinking 'OK, I'm going to go home tonight, am I going to find my mum crying on the floor again drunk?'

For peer review only - http://bmjopen.bmj.com/site/about/guidelines.xhtml

The majority of young people discussed how they have felt "normal" and like part of a family since coming into care. This was expressed regardless of participants' current relationships with biological family, with many describing strong and important bonds with both their biological and foster family. Feeling like part of a family was especially valued by participants and some expressed their appreciation for the support and sense of belonging provided by their foster carers.

Just feel like an ordinary family really, like an ordinary person. Sometimes I don't even realise I'm in care.

I'm lucky I ended up here. I'm so goddamn lucky I ended up here, coz this is my home now, it's my family and I love 'em, I love 'em, I actually do, coz they're my family.

In some cases, these positive aspects were reported to facilitate improvements within different areas of participants' lives, such as at school and when coping with difficult situations and emotions.

They [carers] *like understand what I'm saying and like if I'm annoyed they help me to know how to calm down.*

I started knuckling down at school, because I felt safe.

Subtheme 2: Negative aspects of being in care included feeling different and not being listened to or respected. Most participants discussed feeling "different" because they were a young person in care, and were concerned about others' judgement. Mandatory forms and compulsory meetings with social workers could create difficult situations for participants, or *"annoying"* disruptions, which served as regular reminders that they were different from other children. Alongside this, concerns about others' lack of understanding meant that many participants, adolescents in particular, were very conscious of being judged, particularly within the school context.

Most people think you are naughty 'cause you're in care.

For peer review only - http://bmjopen.bmj.com/site/about/guidelines.xhtml

Like the social workers coming in here [school] or whatever… it's awkward 'cause like a normal person wouldn't really have that.

Amongst adolescents and particularly those struggling with mental health difficulties, feeling different from their peers was often accompanied by complaints of lacking freedom and control, generating a feeling of restricted independence. This led some older participants to express their desire to leave the care system and move into independent living, in order to take back control over their lives.

I have no control over my own life.

I want to go into independent living, because I've had enough of living with, like, parent figures and I just want to be on my own and be independent and prove that I can do it.

Theme 2: Social support was considered key to coping but not always available, while access to professional support was inconsistent. The majority of participants most valued informal, day-to-day support from a trusted adult, while describing formal/professional support to be less available and less helpful. Many participants expressed the need for formal support to be improved and made more readily available.

Subtheme 1: Day-to-day support from a trusted adult was identified as very important for wellbeing. Having a trusted adult to talk to whenever they felt they needed to was identified as the most valuable form of support for participants. Particular aspects valued in a trusted adult were their ability to listen, to *"keep secrets"* and to *"give good advice"*. The importance of being able to call upon this support as and when it was needed was emphasised in discussions. For example, participants described the benefits of being able to talk to carers after a bad day or to speak with key staff at school if there was an argument in a lesson. An important factor in maintaining wellbeing was for participants to know that there was always a trusted adult available to speak with.

Telling someone you trust makes you a lot calmer.

For peer review only - http://bmjopen.bmj.com/site/about/guidelines.xhtml

Overall, carers were by far reported as the primary source of support from the perspective of most of the participants. Some participants also talked about their social worker or a school staff member being key sources of support.

> *..most foster carers are very nice and they really help you with your life… they look after and like you.*
>
> *Whenever something* [is] *tough, I'll tell her* [social worker] *and she'd always take me out and then we'd always chat about something different and then she'd take my mind off it.*
>
> *My school nurse helped me a lot because whenever I had a problem I'd go to her and she'd try to sort it out as quick as possible*

While there were some cases of very positive relationships between a young person and their social worker, perceived ineffective communication between social workers and young people was identified as one of their central problems in terms of feeling supported. A focus of many interviews was how transient their social worker was, with almost all having multiple different social workers over their time in care, or even over the past year. This transience made it particularly difficult for young people to build trust, which could also influence their willingness to disclose information about their early experiences and their general trust of professionals. Some also described situations in which they viewed that confidentiality had been breached by adults in their support network, further resulting in them feeling let down and corroding trust.

> *You don't know who's coming and going.*
>
> *I just want one […] one person [Social Worker] who is going to be there for quite a long time and not going to leave all of a sudden and I can build up a bond.*
>
> *You tell them [about your experiences] and then they leave and you can't tell them anymore. So you're like ohmygod who can I tell? Who can I trust? It's hard.*

For peer review only - http://bmjopen.bmj.com/site/about/guidelines.xhtml

A minority of participants perceived that there was no one they could talk to. These participants were all adolescents and all reported struggling with significant mental health difficulties, including engaging in self-harm and/or drug or alcohol use. For these young people a lack of a trusted adult, which largely included an unstable placement, was particularly problematic. Unhelpful foster placements were discussed in terms of young people perceiving carers fostering *"for the money",* blaming or not trusting the young person, not putting effort in to spending time with them, or the young person perceiving that the carer had not been appropriately trained.

> *Since I was seven all I've ever done is either sat on the sofa and watched tv on my own coz no one talks to me or I've sat in my room and watched tv on my own because nobody ever talks to me*

Subtheme 2: Formal mental health support was often unavailable and opinions on usefulness were mixed. Formal support from mental health professionals was identified as being often unavailable and unhelpful. For those who had seen a mental health professional, this was often the school counsellor, with the minority accessing formal child and adolescent mental health services (CAMHS). From those who had received support, either in the form of a school counsellor or CAMHS, discussions identified mixed opinions with a slight majority describing it as unhelpful. Young people had difficultly specifically identifying what they found unhelpful about this support, although some did describe feeling restricted to a scheduled appointment and having to talk about their past despite not wanting to do so.

> *It made it worse like, having a scheduled time to go and talk about things when I don't really want to talk about them, like I'd rather just say it when I felt like saying it. It just made me think about it more which made it worse.*

For peer review only - http://bmjopen.bmj.com/site/about/guidelines.xhtml

Similarly, those who had positive experiences with mental health professionals struggled to identify what it was that had been helpful, but just that it provided them a safe space to talk and to help them cope.

It made me talk to people and like actually not just keep everything to yourself

I got my feelings clear to everyone

Theme 3: Adaptive and maladaptive coping strategies were used to manage emotional difficulties

When participants were asked what they do when experiencing negative emotions, a range of coping strategies were identified. Some of these strategies were adaptive, such as talking to somebody they trusted, and some were maladaptive, such as self-harm.

Subtheme 1: Talking about worries or challenges was identified as a helpful strategy for coping with negative emotions. Talking about worries or problems with a trusted person was identified as a common strategy used in response to negative emotions, such as sadness, anger, or anxiety. As previously highlighted, carers, teachers and social workers were amongst those who participants felt comfortable speaking to. This was discussed as a primarily helpful strategy, often resulting in feeling better afterwards or enabling problems to be resolved.

If you're feeling down, always talk to someone that you're close to about it, never hold it in.

Subtheme 2: A majority avoided talking about their pre-care experiences and in the context of mental health difficulties maladaptive coping was common. While talking to someone about day-to-day worries was seen as important for coping, most participants thought it was best to avoid talking about their pre-care experiences. Indeed, when asked what advice they might give a friend who was struggling, many endorsed they would

For peer review only - http://bmjopen.bmj.com/site/about/guidelines.xhtml

encourage them to talk to someone, but then also endorsed that this was not something they themselves did.

Speak up and tell people how you are feeling and what you've been through, and that you're struggling to cope. Because if people don't know people can't help.

Interviewer: Did you do that?

No, because I didn't know them and I don't trust people I don't know.

Reasons for avoidance of discussing their maltreatment varied but the most common justifications were either because talking about pre-care experiences made them feel uncomfortable or because they did not think it would be helpful. This sense of discomfort when talking about previous experiences often appeared to be associated with a lack of trust in adults and services. Participants also discussed feeling embarrassed, uncomfortable with the amount of attention placed on them, and unsure of how to act when describing events.

I don't really like talking about my past too much, it's, it's like all attentions on me and it's like awkward.

It don't change nothing, it don't mean it go back. It still happened you can't make it [go away]

From those who identified talking as an unhelpful strategy, many held the attitude that, as events are in the past, talking about events will not change anything. Some also felt that it acted as a barrier to moving forward. This was particularly reported by young people who were also reporting high mental health difficulties, including current distressing intrusive memories of their maltreatment.

I'm not saying "suck it up and be a man" but just try and just keep your mind off it, keep doing what you're doing cos you don't really want to let the past like take over you.

Because the past is in the past and I really don't want to bring my past up. I'm trying to move forward for the minute but obviously if people are bringing it up, I can't.

For peer review only - http://bmjopen.bmj.com/site/about/guidelines.xhtml

In general, many participants endorsed avoidance of maltreatment memories and of talking about their past as a primary way to cope. In most cases this involved distracting themselves with activities, such as watching TV and playing with friends. Indeed in many cases when talking about their support systems, young people endorsed that adults helping to distract them was helpful (e.g changing the topic; social workers taking them to lunch; see Theme 2 quotes). These distractions shifted the focus of their attention onto something else, allowing them to avoid thinking about their early experiences.

[in response to question about coping] *just watch tv or something.*

I would go on the PlayStation and just kill people.

In the case of adolescents who were struggling with mental health difficulties, there were more frequent examples of destructive coping strategies, particularly self-harm and drug and alcohol use. Many of these young people reported that they did not have any other way of coping with their emotional difficulties.

I pretend to be okay when I'm not and let emotions build up until eventually I just break.

Getting drunk a lot, doing a lot of drugs.

I cry and I hurt myself that's literally the only way I know how to deal with things anymore.

Discussion

This study aimed to learn more about the views of young people in out-of-home care, in relation to support and coping with emotional difficulties. Across the interviews there were core common themes, including the importance of having a consistent and trusted adult and the need to feel secure and loved. Carers were identified as the primary source of emotional support by most young people. While many participants talked about the importance of talking to your support system as a means of coping, most also thought that it was not helpful to talk about their pre-care experiences, with avoidant coping strategies common.

For peer review only - http://bmjopen.bmj.com/site/about/guidelines.xhtml

Similar to other qualitative work with young people in care, participants viewed the support of an adult as key to coping with daily stress and interpersonal difficulties, and with more ongoing mental health difficulties (e.g., Davies & Wright, 2008; Sinclair & Wilson, 2003). In the vast majority of cases, the carer (or key worker if in residential care) was viewed as the primary source of this support, although some young people talked about this support also coming from their social worker, a trusted staff member at school, or friend. Trustworthiness was discussed as particularly central to the young person feeling supported, including the perception that the adult would be able to keep information confidential. A lack of trust was often expressed as a key concern and potential barrier to seeking help. While some young people had positive relationships with their social workers, many participants discussed changes in social workers as another key barrier to building a relationship and seeking support for emotional difficulties. This meant for some, although certainly not all, their social worker was not seen as a useful source of support. These findings particularly highlight the need for services to explore policies around mitigating some of the consequences of social worker changes on the young person's wellbeing. Social work has some of the highest burn-out rates of any profession. Providing more robust training and support to social workers will be key to reducing social work changes (Kim et al., 2011). However, given some change is inevitable, policies for how these changes happen are crucial. For example, many young people were not informed that their social worker would change and felt let-down by this change, meaning they had trouble feeling that they could trust the new social worker, who may be gone the next day. Thus, ensuring young people are prepared for the change may be one simple avenue for improving the impact this may have on their wellbeing and perceptions of support.

While many participants talked about actively seeking out their carer for support if they were feeling worried or upset (e.g., if they were upset about something at school), many

For peer review only - http://bmjopen.bmj.com/site/about/guidelines.xhtml

at the same time emphatically stated that they did not think it was useful to talk about their maltreatment experiences, with either their carer or mental health professionals. While this was the case across much of the sample, it is perhaps most concerning for those young people who discussed experiencing significant mental health difficulties. This sub-set of the sample most commonly reported feeling that they had no person they could turn to for support, that they did not want to talk to a professional about their maltreatment, and had no strategies for coping with their mental health beyond self-harm and drug and alcohol abuse. Beliefs around not talking about maltreatment or trauma present a particular challenge to mental health and social-care professionals. While it is understandable that young people may not want to talk about distressing experiences, avoiding talking or thinking about traumatic experiences (i.e., thought suppression) is considered a key maintainer of poor mental health outcomes (e.g., Trickey et al., 2012). Further, a key component of evidenced-based trauma-focused psychological support (i.e. trauma-focused cognitive and behavioural therapy) involves processing the traumatic experiences, commonly via a trauma narrative (e.g., see Deblinger et al., 2011). There remains an urgent need to understand how services can best engage young people in care in psychological treatments that might involve talking about their experiences, particularly for a group where developing trust and a strong therapeutic relationship is essential (Shaw & de Jong, 2012). This may be partly achieved by having systems, including at a social-care and education level, where staff are appropriately trained and supported in engaging with these challenging conversations with young people from earlier on in their time in care, so young people know that it is safe to talk about these experiences if they want to.

While only the minority of participants in this study had received professional mental health support, including via CAMHS and school counselling, views on this support were also mixed. In their 2008 review of qualitative literature on children in care and mental health

For peer review only - http://bmjopen.bmj.com/site/about/guidelines.xhtml

services, Davies and Wright highlighted that many young people felt wary and unsure of professional mental health support, and highlighted the importance of developing trust. Ten years later, the current study found that young people in care are still experiencing these same concerns. While some young people discussed strong relationships with mental health professionals and benefits to this support, many expressed concerns with being expected to talk about their experiences, as well as concerns around stigma, particularly if they were asked to see a school counsellor (i.e., where their peers would potentially know), as barriers to benefiting from this support. There remains an urgent need to improve understanding of the service-level barriers to accessing professional support, including access to evidence-based psychological interventions. Within the current UK CAMHS system, thresholds for professional mental health support often mean young people are in crisis, with complex mental health needs, by the time they can access support (e.g., see Blow, 2015). Exploring feasible avenues for providing effective evidence-based support, before reaching crisis, is clearly an important avenue for addressing the mental health needs of these young people.

While this study has key strengths, including a focus on an under-researched group, and the use of qualitative interviews to provide an in-depth exploration of their views on coping and wellbeing, findings should also be considered in light of key limitations. First, we used opportunity sampling methods. All young people were participants in a larger quantitative study of mental health and wellbeing. Young people who did not want to talk about their experiences and mental health would likely have declined participating in this study. Participants also all came from two Local Authorites in England. While findings cannot necessarily be generalised to other young people in out-of-home care, it is worth noting the significant overlap between these findings and qualitative work with young people in care and foster carers in the UK, US, and Australia (e.g., Ahrens et al., 2011; Davies & Wright, 2008). Finally, quantitative research is needed to provide more robust evidence on

For peer review only - http://bmjopen.bmj.com/site/about/guidelines.xhtml

the consequences of different support systems and coping strategies in relation to the mental health of these young people.

In sum, while there were positives to being in care and many young people felt well supported by their carer, results of this qualitative study identified key areas to be addressed to improve support for the emotional wellbeing of this group. This includes services exploring policy around social worker changes, ensuring foster carers are appropriately trained to support young people, and ensuring young people are receiving evidence-based psychological support in a more timely manner, before reaching crisis point. Addressing these issues remains a significant challenge, but the current work found failing to provide adequate support, especially for those with the most significant mental health difficulties, had major consequences for the young person's wellbeing.

For peer review only

For peer review only - http://bmjopen.bmj.com/site/about/guidelines.xhtml

Contributors: RMH is the lead researcher on the project and PI on the ESRC grant. She led the conceptualisation of the project, conducted a proportion of the interviews, and was responsible for overall project management. SLH was involved in the conceptualisation of the project, including the design of the interview schedule and also contributed to discussion on the themes and feedback on the manuscript. RM-S was involved in the conceptualisation of the project and contributed to the discussion about the themes and feedback on the manuscript. EE was the research assistant on the project and conducted a proportion of the interviews and contributed to transcribing, data analysis, and feedback on the manuscript. ER-E was involved in the transcribing, contributed to data analysis and provided feedback on the manuscript. TH contributed to transcribing and feedback on the codes and themes.

The authors also wish to sincerely thank the many social-care and mental health service staff, carers, and young people who contributed to the design of this project and from whom we continue to learn so much.

Funding: This research was funded by an ESRC Future Leader Grant (ES/N01782X/1) and University of Bath Prize Fellowship awarded to RMH.

Competing Interests: None to declare.

Data sharing: Anonymised transcripts will be available on request on a case-by-case basis.

For peer review only - http://bmjopen.bmj.com/site/about/guidelines.xhtml

References

Ahrens, K. R., DuBois, D. L., Garrison, M., Spencer, R., Richardson, L. P., & Lozano, P. (2011). Qualitative exploration of relationships with important non-parental adults in the lives of youth in foster care. *Children and Youth Services Review*, *33*(6), 1012-1023.

Blow, E. (2015) *Children and young people's mental health – policy, CAMHS services, funding and education*. London: House of Commons Library.

Braun, V., & Clarke, V. (2006). Using thematic analysis in psychology. *Qualitative research in Psychology*, *3*(2), 77-101.

Davies, J., & Wright, J. (2008). Children's voices: A review of the literature pertinent to looked-after children's views of mental health services. *Child and Adolescent Mental Health*, *13*(1), 26-31.

Deblinger, E., Mannarino, A. P., Cohen, J. A., Runyon, M. K., & Steer, R. A. (2011). Trauma-focused cognitive behavioral therapy for children: impact of the trauma narrative and treatment length. *Depression and Anxiety*, *28*(1), 67-75.

Department for Education. (2018). *Statistics: looked after children*. London: Author.

Docherty, S., & Sandelowski, M. (1999). Focus on qualitative methods: Interviewing children. *Research in Nursing & Health*, *22*(2), 177-185.

Ford, T., Vostanis, P., Meltzer, H., & Goodman, R. (2007). Psychiatric disorder among British children looked after by local authorities: comparison with children living in private households. *The British Journal of Psychiatry*, *190*(4), 319-325.

Hiller, R. M., & St Clair, M. C. S. (2018). The emotional and behavioural symptom trajectories of children in long-term out-of-home care in an English local authority. *Child Abuse & Neglect*, *81*, 106-117.

Hiller, R. M., Meiser-Stedman, R., Elliott, E., Banting, R., Halligan, S.L. (2020). Cognitive

For peer review only - http://bmjopen.bmj.com/site/about/guidelines.xhtml

predictors of (complex) posttraumatic stress: A longitudinal study of young people in out-of-home care. *Journal of Child Psychology and Psychiatry.* In Press.

Jones, R., Everson-Hock, E. S., Papaioannou, D., Guillaume, L., Goyder, E., Chilcott, J., ... & Swann, C. (2011). Factors associated with outcomes for looked-after children and young people: a correlates review of the literature. *Child: Care, Health and Development*, *37*(5), 613-622.

Kim, H., Ji, J., & Kao, D. (2011). Burnout and physical health among social workers: A three-year longitudinal study. *Social Work*, *56*(3), 258-268.

McMillen, J. C., Zima, B. T., Scott Jr, L. D., Auslander, W. F., Munson, M. R., Tollie, M. T., Spitznagel, E. L. (2005). Prevalence of psychiatric disorders among older youths in the foster care system. *Journal of the American Academy of Child & Adolescent Psychiatry*, *44*(1), 88-95.

Murray, L., Creswell, C., & Cooper, P. J. (2009). The development of anxiety disorders in childhood: an integrative review. *Psychological Medicine*, *39*(9), 1413-1423.

Newton, R. R., Litrownik, A. J., & Landsverk, J. A. (2000). Children and youth in foster care: Disentangling the relationship between problem behaviors and number of placements. *Child Abuse & Neglect*, *24*(10), 1363-1374.

Oswald, S. H., Heil, K., & Goldbeck, L. (2009). History of maltreatment and mental health problems in foster children: A review of the literature. *Journal of Pediatric Psychology*, *35*(5), 462-472.

Pecora, P. J., White, C. R., Jackson, L. J., & Wiggins, T. (2009). Mental health of current and former recipients of foster care: A review of recent studies in the USA. *Child & Family Social Work*, *14*(2), 132-146.

Rock, S., Michelson, D., Thomson, S., & Day, C. (2013). Understanding foster placement

For peer review only - http://bmjopen.bmj.com/site/about/guidelines.xhtml

instability for looked after children: A systematic review and narrative synthesis of quantitative and qualitative evidence. *British Journal of Social Work*, *45*(1), 177-203.

Sachser, C., Berliner, L., Holt, T., Jensen, T. K., Jungbluth, N., Risch, E., ... & Goldbeck, L. (2017). International development and psychometric properties of the Child and Adolescent Trauma Screen (CATS). *Journal of Affective Disorders*, *210*, 189-195.

Shaw, M., & De Jong, M. (2012). Child abuse and neglect: a major public health issue and the role of child and adolescent mental health services. *The Psychiatrist*, *36*(9), 321-325.

Sinclair, I., & Wilson, K. (2003). Matches and mismatches: The contribution of carers and children to the success of foster placements. *British Journal of Social Work*, *33*(7), 871-884.

Trickey, D., Siddaway, A. P., Meiser-Stedman, R., Serpell, L., & Field, A. P. (2012). A meta-analysis of risk factors for post-traumatic stress disorder in children and adolescents. *Clinical Psychology Review*, *32*(2), 122-138.

Van Wesel, F., Boeije, H., Alisic, E., & Drost, S. (2012). I'll be working my way back: A qualitative synthesis on the trauma experience of children. *Psychological Trauma: Theory, Research, Practice, and Policy*, *4*(5), 516.

Vasileiou, K., Barnett, J., Thorpe, S., & Young, T. (2018). Characterising and justifying sample size sufficiency in interview-based studies: systematic analysis of qualitative health research over a 15-year period. *BMC Medical Research Methodology*, *18*(1), 148.

For peer review only - http://bmjopen.bmj.com/site/about/guidelines.xhtml

Table 1. *Sample demographics.*

**Young person descriptives (N = 25)**	
Age, *M* (*SD*), Range	13.0 (2.24)
Sex (% female)	56%
Ethnicity (% Caucasian)	80%
Age entered care, *M* (*SD*)	8.7 yrs (4.2)
Time in care, *M* (*SD*)	4.2 yrs (3.6)
% who had multiple placements	80%
Type of placement	
Non-biological foster carer	76%
Biological foster carer (e.g., grandmother)	16%
Residential care home	8%

For peer review only

For peer review only - http://bmjopen.bmj.com/site/about/guidelines.xhtml

Table 2. *Themes and subthemes from thematic analysis.*

Themes	Sub-themes
**Being in care has positive and negative aspects.**	Being in care can create a sense of safety and of being part of a family.
	Negative aspects of being in care included feeling different and not being listened to or respected.
**Social support was considered key to coping but not always available, while access to professional support was inconsistent.**	Day-to-day support from a trusted adult was identified as very important for wellbeing.
	Formal mental health support was often unavailable and opinions on usefulness were mixed.
**Adaptive and maladaptive coping strategies were used to manage emotional difficulties.**	Talking about worries or challenges was identified as a helpful strategy for coping with negative emotions.
	A majority avoided talking about their pre-care experiences and in the context of mental health difficulties maladaptive coping was common.

For peer review only - http://bmjopen.bmj.com/site/about/guidelines.xhtml

Reporting checklist for qualitative study.

Based on the SRQR guidelines.

Instructions to authors

Complete this checklist by entering the page numbers from your manuscript where readers will find each of the items listed below.

Your article may not currently address all the items on the checklist. Please modify your text to include the missing information. If you are certain that an item does not apply, please write "n/a" and provide a short explanation.

Upload your completed checklist as an extra file when you submit to a journal.

In your methods section, say that you used the SRQRreporting guidelines, and cite them as:

O'Brien BC, Harris IB, Beckman TJ, Reed DA, Cook DA. Standards for reporting qualitative research: a synthesis of recommendations. Acad Med. 2014;89(9):1245-1251.

		Reporting Item	Page Number
**Title**			
	#1	Concise description of the nature and topic of the study identifying the study as qualitative or indicating the approach (e.g. ethnography, grounded theory) or data collection methods (e.g. interview, focus group) is recommended	1

For peer review only

Abstract

	#2	Summary of the key elements of the study using the abstract format of the intended publication; typically includes background, purpose, methods, results and conclusions	2

Introduction

Problem formulation	#3	Description and signifcance of the problem / phenomenon studied: review of relevant theory and empirical work; problem statement	4-5
Purpose or research question	#4	Purpose of the study and specific objectives or questions	5

Methods

Qualitative approach and research paradigm	#5	Qualitative approach (e.g. ethnography, grounded theory, case study, phenomenolgy, narrative research) and guiding theory if appropriate; identifying the research paradigm (e.g. postpositivist, constructivist / interpretivist) is also recommended; rationale. The rationale should briefly discuss the justification for choosing that theory, approach, method or technique rather than other options available; the assumptions and limitations implicit in those choices and how those choices influence study conclusions and transferability.	7-8

For peer review only

		As appropriate the rationale for several items might be discussed together.	
Researcher characteristics and reflexivity	#6	Researchers' characteristics that may influence the research, including personal attributes, qualifications / experience, relationship with participants, assumptions and / or presuppositions; potential or actual interaction between researchers' characteristics and the research questions, approach, methods, results and / or transferability	7
Context	#7	Setting / site and salient contextual factors; rationale	6
Sampling strategy	#8	How and why research participants, documents, or events were selected; criteria for deciding when no further sampling was necessary (e.g. sampling saturation); rationale	6
Ethical issues pertaining to human subjects	#9	Documentation of approval by an appropriate ethics review board and participant consent, or explanation for lack thereof; other confidentiality and data security issues	6
Data collection methods	#10	Types of data collected; details of data collection procedures including (as appropriate) start and stop dates of data collection and analysis, iterative process, triangulation of sources / methods, and modification of procedures in response to evolving study findings; rationale	6

For peer review only

Data collection instruments and technologies	#11	Description of instruments (e.g. interview guides, questionnaires) and devices (e.g. audio recorders) used for data collection; if / how the instruments(s) changed over the course of the study	6-8
Units of study	#12	Number and relevant characteristics of participants, documents, or events included in the study; level of participation (could be reported in results)	6-8
Data processing	#13	Methods for processing data prior to and during analysis, including transcription, data entry, data management and security, verification of data integrity, data coding, and anonymisation / deidentification of excerpts	6-8
Data analysis	#14	Process by which inferences, themes, etc. were identified and developed, including the researchers involved in data analysis; usually references a specific paradigm or approach; rationale	6-8
Techniques to enhance trustworthiness	#15	Techniques to enhance trustworthiness and credibility of data analysis (e.g. member checking, audit trail, triangulation); rationale	7-8
**Results/findings**			
Syntheses and interpretation	#16	Main findings (e.g. interpretations, inferences, and themes); might include development of a theory or model, or integration with prior research or theory	8-16

For peer review only

Links to empirical data	#17	Evidence (e.g. quotes, field notes, text excerpts, photographs) to substantiate analytic findings	8-16
**Discussion**			
Intergration with prior work, implications, transferability and contribution(s) to the field	#18	Short summary of main findings; explanation of how findings and conclusions connect to, support, elaborate on, or challenge conclusions of earlier scholarship; discussion of scope of application / generalizability; identification of unique contributions(s) to scholarship in a discipline or field	16
Limitations	#19	Trustworthiness and limitations of findings	19
**Other**			
Conflicts of interest	#20	Potential sources of influence of perceived influence on study conduct and conclusions; how these were managed	21
Funding	#21	Sources of funding and other support; role of funders in data collection, interpretation and reporting	21

Notes:

- 21: n/a (blinded) The SRQR checklist is distributed with permission of Wolters Kluwer © 2014 by the Association of American Medical Colleges. This checklist was completed on 30. July 2019 using https://www.goodreports.org/, a tool made by the EQUATOR Network in collaboration with Penelope.ai

For peer review only

Coping and support-seeking in out-of-home care: A qualitative study of the views of young people in care in England

Journal:	*BMJ Open*
Manuscript ID	bmjopen-2020-038461.R1
Article Type:	Original research
Date Submitted by the Author:	25-Jul-2020
Complete List of Authors:	Hiller, Rachel; University of Bath, Department of Psychology<br>Halligan, Sarah; University of Bath, Psychology; University of Cape Town, Department of Psychiatry and Mental Health<br>Meiser-Stedman , Richard ; University of East Anglia,<br>Elliott, Elizabeth; University of Bath, Department of Psychology; University of Reading, School of Psychology and Clinical Language Science<br>Rutter-Eley, Emily; University of Reading, School of Psychology and Clinical Language Science<br>Hutt, Tilly; University of Bath, Department of Psychology
**Primary Subject Heading**:	Mental health
Secondary Subject Heading:	Qualitative research, Paediatrics
Keywords:	Child protection < PAEDIATRICS, Child & adolescent psychiatry < PSYCHIATRY, Quality in health care < HEALTH SERVICES ADMINISTRATION & MANAGEMENT

SCHOLARONE™
Manuscripts

I, the Submitting Author has the right to grant and does grant on behalf of all authors of the Work (as defined in the below author licence), an exclusive licence and/or a non-exclusive licence for contributions from authors who are: i) UK Crown employees; ii) where BMJ has agreed a CC-BY licence shall apply, and/or iii) in accordance with the terms applicable for US Federal Government officers or employees acting as part of their official duties; on a worldwide, perpetual, irrevocable, royalty-free basis to BMJ Publishing Group Ltd ("BMJ") its licensees and where the relevant Journal is co-owned by BMJ to the co-owners of the Journal, to publish the Work in this journal and any other BMJ products and to exploit all rights, as set out in our licence.

The Submitting Author accepts and understands that any supply made under these terms is made by BMJ to the Submitting Author unless you are acting as an employee on behalf of your employer or a postgraduate student of an affiliated institution which is paying any applicable article publishing charge ("APC") for Open Access articles. Where the Submitting Author wishes to make the Work available on an Open Access basis (and intends to pay the relevant APC), the terms of reuse of such Open Access shall be governed by a Creative Commons licence – details of these licences and which Creative Commons licence will apply to this Work are set out in our licence referred to above.

Other than as permitted in any relevant BMJ Author's Self Archiving Policies, I confirm this Work has not been accepted for publication elsewhere, is not being considered for publication elsewhere and does not duplicate material already published. I confirm all authors consent to publication of this Work and authorise the granting of this licence.

For peer review only

Coping and support-seeking in out-of-home care: A qualitative study of the views of young people in care in England

Rachel M Hiller[1], Sarah L Halligan[1,2], Richard Meiser-Stedman[3], Elizabeth Elliott[1,4], Emily Rutter-Eley[4], Tilly Hutt[1]

[1] Department of Psychology, University of Bath, UK

[2] Department of Psychiatry and Mental Health, University of Cape Town, South Africa

[3] Department of Clinical Psychology, Norwich Medical School, University of East Anglia, UK

[4] School of Psychology and Clinical Language Science, University of Reading, UK

* Corresponding Author:

Dr Rachel Hiller

Department of Psychology

University of Bath

P. 01225 383794

E. R.Hiller@bath.ac.uk

Main text word count: 5, 530

For peer review only

Abstract

Objectives: Young people who have been removed from their family home and placed in out-of-home care have commonly experienced abuse, neglect and/or other forms of early adversity. High rates of mental health difficulties have been well-documented in this group. The aim of this research was to explore the experiences of these young people within the care system, particularly in relation to support-seeking and coping with emotional needs, to better understand feasible and acceptable ways to improve outcomes for this group.

Design and Study-Setting: This study used 1:1 semi-structured qualitative interviews with young people in out-of-home care in England, to provide an in-depth understanding of their views of coping and support for their emotional needs, both in terms of support networks and experiences with mental health services. Participants were 25 10-16 years olds (56% female) and included young people living with non-biological foster carers, kinship carers, and in residential group homes.

Results: Participants described positive (e.g. feeling safe) and negative (e.g. feeling judged) aspects to being in care. Carers were identified as the primary source of support, with a supportive adult central to coping. Views on support and coping differed for young people who were experiencing more significant mental health difficulties, with this group largely reporting feeling unsupported and many engaging in self-harm. The minority of participants had accessed formal mental health support, and opinions on usefulness were mixed.

Conclusions: Results provide insight, from the perspective of care-experienced young people, about both barriers and facilitators to help-seeking, as well as avenues for improving support.

Key words: child maltreatment; care-experienced; adolescents; foster care; emotional wellbeing; mental health; help-seeking; qualitative

For peer review only

Strengths and Limitations of the Study:

- This study is focused on young people living in out-of-home care, representing an under-researched group where there remains significant need.
- We used qualitative interview methods to gain in-depth understanding of the views and experiences of these young people.
- The study recruited from two English Local Authorities.
- This study adds to the limited literature on how young people in care cope and seek support when they experience emotional difficulties.
- As this study uses qualitative methods, we cannot conclude whether different types of reported coping might be associated with better or worse outcomes.

For peer review only

For peer review only - http://bmjopen.bmj.com/site/about/guidelines.xhtml

Introduction

The most common reason a child would be removed from their home and placed in out-of-home care is due to the experience of abuse and/or neglect [e.g., 1,2]. In the UK, it is also the case that most young people who enter care are removed from them home when school-aged or older, meaning early experiences of maltreatment have often been ongoing for a number of years [1]. Once in care many continue to face ongoing instability. Separation from siblings is common, as are moves between different placement providers, resulting in the potential for inconsistent support [3-4]. Given these accumulative experiences, it is perhaps unsurprising that there is well-established evidence that young people in out-of-home care experience high rates of psychological difficulties compared to their peers [5-7].

Failing to adequately address the psychology or emotional needs of young people in care has been identified as a key driver of many of the well-documented poor outcomes that can categorise this group, including school attainment and later rates of homelessness and unemployment [8]. Yet, despite the long-documented poor outcomes, there has been limited evidence of improvements in the overall picture of mental health for children in care in the UK over the past decade [1]. One concern is that these young people can fall between the gaps of social-care and national health service provisions, which can result in poor access to mental health services [9-10]. With resource issues affecting child and adolescent mental health services (CAMHS) in many countries, it is also largely impossible for these services to address the large number of young people in care who may benefit from professional mental health support.

In the face of potentially limited professional support services, young people will inevitably develop day-to-day coping strategies and support networks – whether perceived to be adaptive or maladaptive. Some qualitative work has investigated the views of young people in care in relation to support networks, and specifically their relationships with carers,

For peer review only - http://bmjopen.bmj.com/site/about/guidelines.xhtml

finding developing trust, consistent boundaries, and empathy were viewed as important [11-12]. However, there is less evidence for how they might engage with other support networks, including peers, school, and social workers. Such evidence is potentially particularly important for this group, given one-third of young people in care in the UK will have more than one carer in any given year [1], and given the well-documented cyclical association between placement breakdowns and emotional difficulties [4,13]. Beyond the role of external support networks, we know little about what strategies young people in care may be using themselves to manage their emotional wellbeing. Understanding how young people in care cope with emotional difficulties, particularly in the face of potentially disrupted support networks, is crucial to understanding the experiences of this group and avenues for improving formal and informal support, particularly given evidence from the wider child mental health field that certain types of coping, especially avoidance, can exacerbate mental health difficulties [14-15], including in care-experienced young people [16].

Using a semi-structured qualitative approach we sought to explore the following questions: (1) where do young people in care seek support for emotional difficulties, both in terms of social support and professional services?; (2) what do they view as barriers to seeking help?; and (3) what coping strategies do they use when experiencing emotional difficulties? This included a focus on more day-to-day coping and help-seeking (e.g., if they felt stressed) and coping and help-seeking around more significant mental health difficulties.

Method

Recruitment and Sample

Ethical approval was granted from the University of Bath Psychology- and Social Care- research ethics committees, along with permission from the participating local authorities. Using opportunity sampling, we recruited 25 young people aged 10 – 16 years old. Participants were all part of a larger quantitative study on the mental health and

For peer review only - http://bmjopen.bmj.com/site/about/guidelines.xhtml

wellbeing of young people in care [16]. The sample size is based on general guidance for qualitative methodology [see 17-18]. While clear guidance for sample sizes in qualitative research remains limited, our sample size was chosen to be in line with, or larger than, similar studies in the field [e.g., 19-20]. It was also based on our previous qualitative work with young people in terms of the sample required to reach saturation, with capacity to continue recruitment if it was felt saturation was not reached or the data was inadequate (which was ultimately not the case). The sample size also allowed us to explore views of older and younger children within our age range, and include young people with a range of mental health needs, from those who appeared to be managing well to those struggling with more chronic mental health difficulties.

Participants came from two local authorities in England. One was a smaller-sized local authority and one was medium-sized, with both in largely urban areas. Informed consent for the young person's participation was provided by their social worker, while the young person provided informed assent (or consent if 16 years old). Eligibility for participation in the quantitative study were: aged 10-17 years old, on full care order if under 16 years old (where the local authority has acquired parental responsibility for the young person), absence of developmental disability that would preclude the child from mainstream schooling, and absence of significant current risk (e.g., current severe suicidality). Young people could be in any style of placement, including foster placement with a non-biological carer, kinship placement, residential care home. Descriptive information on the sample is presented in Table 1.

Procedure and Measures

In-person 1:1 interviews were conducted by two researchers. The lead researcher (RH) had prior training and experience in qualitative research, while the other (EE) was experienced researching with vulnerable young people and adults and received further

For peer review only

training specifically on qualitative procedures. We used a 1:1 semi-structured interview format, rather than focus group, due to the potential for sensitive information being discussed that young people may not wish to disclose in a group. For one of these interviews two siblings requested to complete the interview together, while for two interviews the carer remained present at the request of the young person. All others (*n* = 22) had only the young person and interviewer present. RH and EE met regularly throughout the study to monitor the quality of the interviews and reflect on the content. All interviews were audio recorded and later transcribed verbatim. As a thank you for volunteering their time, the young people each received a £10 voucher. As per standard risk management and safeguarding procedures, where appropriate, summary letters were provided to social workers to support potential referrals to mental health services.

Descriptives and semi-structured interview. Basic descriptive information was collected from the young person's social worker, as part of the larger quantitative study. This included the age and reason that they entered care. The semi-structured interview was developed based on the literature on qualitative interviewing techniques and qualitative and quantitative work in the child mental health field [e.g., 9, 21-22]. Interviews broadly covered four core areas: (i) views on what is broadly helpful and unhelpful within the care system, (ii) coping and support for children's mental health and wellbeing (including at home, within social-care, and experiences around mental health services), (iii) coping and support for trauma-related mental health more specifically (e.g., how young people cope with memories of their maltreatment), and (iv) views on ideal support. Questions were primarily focused on the young person's own experiences (see supplementary material for interview schedule). Additionally, participants were also asked what advice they might give a friend in a similar situation, to elicit broader information from young people who may struggle to answer these questions about themselves. Questions were primarily open-ended (e.g., "If you're having a

For peer review only

hard time what do you do to make yourself feel better or different?"). Some direct prompting and questioning was used to clarify comments and fain further insight (e.g., "Was that helpful?"). Interviews lasted from 6-30 minutes.

Data Analyses

Transcripts were analysed using a reflexive thematic analysis approach [23-24] in NVivo 12. Given the exploratory nature of the study, thematic analysis was chosen as the data analytic approach as it allows for a detailed exploration of the data and does not rely on a specific theoretical framework. Co-author (ER), who was not involved in any of the interviews or the broader quantitative study, coded all transcripts. While the purpose of qualitative research is not to completely remove or minimise the influence of researcher subjectivity, a coder who was had not been involved in study design or data collection was used, to reduce potential biases. She first read all transcripts to gain an understanding of the overall data (data immersion) and then systematically coded each transcript using an iterative process [24]. Following the systematic coding of all transcripts, codes were grouped to form overarching themes. The broader themes were also separated into subthemes, to accurately capture more fine grain information provided by participants and ensure the themes appropriately reflected different viewpoints. Co-authors (TH) independently explored all (un-coded) transcripts and generated their own key themes. A consensus meeting showed strong agreement between the primary themes. There was not capacity within the study to seek further input from participants at this point, however reflective practices were used throughout all interviews to ensure clear understanding of the discussion and to minimise any chance of misinterpreting what was being said (e.g., reflecting, summarising back to participants and seeking further clarification where needed).

Patient and Public Involvement

For peer review only - http://bmjopen.bmj.com/site/about/guidelines.xhtml

This study occurred as part of a series of projects, which involves extensive consultation with services, as well as feedback from care-experienced young people and foster carers. This feedback particularly shaped the research questions and use of qualitative methods. Young people and carers were not involved in recruitment for the project. There has been wide written and verbal feedback to stakeholders as part of the larger project.

Results

Descriptive Information

The sample comprised of 14 females and 11 males, aged 10 to 16 years old. They had entered care between infancy and 16 years old, and had been in care from 6 months to almost 14 years, while 80% of the sample had had more than one placement since entering care. Most (76%) were living in a foster placement with a non-biological carer, while four were in a kinship placement (e.g., with a grandmother), and two lived in residential care homes. All were in care due to the experience of abuse and/or neglect.

Interview Themes

Thematic analysis identified three primary themes covering the broader experience of being in care (Theme 1); the centrality of social support to wellbeing and mixed views on professional help (Theme 2); and the use of both adaptive and maladaptive day-to-day coping strategies (Theme 3). A list of themes and subthemes can be found in Table 2.

Theme 1: Being in care could create a sense of safety but some young people struggled with a lack of control and agency

When asked what it was like to be in care, most, but not all participants expressed that being in care was a better or safer alternative to living with their biological family and that they felt safer in care. However, almost all discussed occasions of feeling stigmatised, as well as experiences of being poorly supported by services.

For peer review only

Subtheme 1: Being in care can create a sense of safety and of being part of a family. The majority of participants reported that feeling safe and secure was a particularly positive aspect of being in care. Some participants also described experiencing a sense of relief when first coming into care, recognising that it was not appropriate for them to remain in an unsafe environment.

Relieved actually from going to a bad environment into somewhere I thought could be quite safe.

To know at the end of the day you can go home, instead of thinking 'OK, I'm going to go home tonight, am I going to find my mum crying on the floor again drunk?'

The majority of young people discussed how they have felt "normal" and like part of a family since coming into care. This was expressed regardless of participants' current relationships with biological family, with many describing strong and important bonds with both their biological and foster family. Feeling like part of a family was especially valued and some expressed their appreciation for the support and sense of belonging provided by their foster carers.

Just feel like an ordinary family really, like an ordinary person. Sometimes I don't even realise I'm in care.

I'm lucky I ended up here. I'm so goddamn lucky I ended up here, coz this is my home now, it's my family and I love 'em, I love 'em, I actually do, coz they're my family.

In some cases, these positive aspects were reported to facilitate improvements within different areas of participants' lives, such as at school and when coping with difficult situations and emotions.

They [carers] *like understand what I'm saying and like if I'm annoyed they help me to know how to calm down.*

I started knuckling down at school, because I felt safe.

For peer review only - http://bmjopen.bmj.com/site/about/guidelines.xhtml

Subtheme 2: Negative aspects of being in care included feeling different and not being listened to or respected. Most participants discussed feeling "different" because they were a young person in care, and were concerned about others' judgement. Mandatory forms and compulsory meetings with social workers could create difficult situations for participants, or *"annoying"* disruptions, which served as regular reminders that they were different from other children. Alongside this, concerns about others' (e.g., teachers, peers) lack of understanding meant that many participants, adolescents in particular, were very conscious of being judged, particularly within the school context.

Most people think you are naughty 'cause you're in care.

Like the social workers coming in here [school] or whatever… it's awkward 'cause like a normal person wouldn't really have that.

Amongst adolescents and particularly those struggling with mental health difficulties, feeling different from their peers was often accompanied by complaints of lacking freedom and control, generating a feeling of restricted independence. This led some older participants to express their desire to leave the care system and move into independent living, in order to take back control over their lives.

I have no control over my own life.

I want to go into independent living, because I've had enough of living with, like, parent figures and I just want to be on my own and be independent and prove that I can do it.

Theme 2: Social support was considered key to coping but not always available, while access to professional support was inconsistent. The majority of participants most valued informal, day-to-day support from a trusted adult, while describing formal/professional support to be less available and less helpful. Many participants expressed the need for professional support to be improved and made more readily available.

For peer review only - http://bmjopen.bmj.com/site/about/guidelines.xhtml

Subtheme 1: Day-to-day support from a trusted adult was important for wellbeing.

Having a trusted adult to talk to whenever they felt they needed to was identified as the most valuable form of support for participants. Particular aspects valued in a trusted adult were their ability to listen, to *"keep secrets"* and to *"give good advice"*. The importance of being able to call upon this support as and when it was needed was often emphasised. For example, participants described the benefits of being able to talk to carers after a bad day or to speak with key staff at school if there was an argument in a lesson. Knowing there was always a trusted adult available to speak to was central to maintaining wellbeing for many participants.

Telling someone you trust makes you a lot calmer.

Overall, carers were by far reported as the primary source of support from the perspective of most of the participants. Some participants also talked about their social worker or a school staff member being key sources of support.

..most foster carers are very nice and they really help you with your life… they look after and like you.

Whenever something [is] *tough, I'll tell her* [social worker] *and she'd always take me out and then we'd always chat about something different and then she'd take my mind off it.*

My school nurse helped me a lot because whenever I had a problem I'd go to her and she'd try to sort it out as quick as possible

While there were some cases of very positive relationships between a young person and their social worker, perceived ineffective communication between social workers and young people was identified as one of their central problems in terms of feeling supported. A focus of many interviews was how transient their social worker was, with almost all having multiple different social workers over their time in care, or even over the past year. This transience made it particularly difficult for young people to build trust, which could also influence their willingness to disclose information about their early experiences and their

For peer review only - http://bmjopen.bmj.com/site/about/guidelines.xhtml

general trust of professionals. Some also described situations in which they viewed that confidentiality had been breached by adults in their support network, further resulting in them feeling let down and corroding trust.

You don't know who's coming and going.

I just want one […] one person [Social Worker] who is going to be there for quite a long time and not going to leave all of a sudden and I can build up a bond.

You tell them [about your experiences] and then they leave and you can't tell them anymore. So you're like ohmygod who can I tell? Who can I trust? It's hard.

A minority of participants perceived that there was no one they could talk to. These participants were all adolescents and all reported struggling with significant mental health difficulties, including engaging in self-harm and/or drug or alcohol use. For these young people a lack of a trusted adult, which largely included an unstable placement, was particularly problematic. Unhelpful foster placements were discussed in terms of young people perceiving carers fostering *"for the money"*, blaming or not trusting the young person, not putting effort in to spending time with them, or the young person perceiving that the carer had not been appropriately trained.

Since I was seven all I've ever done is either sat on the sofa and watched tv on my own coz no one talks to me or I've sat in my room and watched tv on my own because nobody ever talks to me

Subtheme 2: Formal mental health support was often unavailable and opinions on usefulness were mixed. Formal support from mental health professionals was identified as being often unavailable and unhelpful. For those who had seen a mental health professional, this was often the school counsellor, with the minority accessing formal child and adolescent mental health services (CAMHS). From those who had received support, either in the form of a school counsellor or CAMHS, discussions identified mixed opinions with a slight majority

For peer review only - http://bmjopen.bmj.com/site/about/guidelines.xhtml

describing it as unhelpful. Young people had difficultly specifically identifying what they found unhelpful about this support, although some did describe feeling restricted to a scheduled appointment and having to talk about their past despite not wanting to do so.

It made it worse like, having a scheduled time to go and talk about things when I don't really want to talk about them, like I'd rather just say it when I felt like saying it.

It just made me think about it more which made it worse.

Similarly, those who had positive experiences with mental health professionals struggled to identify what it was that had been helpful, but just that it provided them a safe space to talk and to help them cope.

It made me talk to people and like actually not just keep everything to yourself

I got my feelings clear to everyone

Theme 3: Talking about problems was seen as important, but maladaptive coping strategies were also reported.

When participants were asked what they do when experiencing negative emotions, a range of coping strategies were identified. Some of these strategies were adaptive, such as talking to somebody they trusted, and some were maladaptive, such as self-harm.

Subtheme 1: Talking about worries or challenges was identified as a helpful strategy for coping with negative emotions. Talking about worries or problems with a trusted person was identified as a common strategy used in response to negative emotions, such as sadness, anger, or anxiety. As previously highlighted, carers, teachers and social workers were amongst those who participants felt comfortable speaking to. This was discussed as a primarily helpful strategy, often resulting in feeling better afterwards or enabling problems to be resolved.

If you're feeling down, always talk to someone that you're close to about it, never hold it in.

For peer review only - http://bmjopen.bmj.com/site/about/guidelines.xhtml

Subtheme 2: A majority avoided talking about their pre-care experiences and in the context of mental health difficulties maladaptive coping was common. While talking to someone about day-to-day worries was seen as important for coping, most participants thought it was best to avoid talking about their pre-care experiences. Indeed, when asked what advice they might give a friend who was struggling, many endorsed they would encourage them to talk to someone, but then also endorsed that this was not something they themselves did.

Speak up and tell people how you are feeling and what you've been through, and that you're struggling to cope. Because if people don't know people can't help.

Interviewer: Did you do that?

No, because I didn't know them and I don't trust people I don't know.

Reasons for avoidance of discussing their maltreatment varied but the most common justifications were either because talking about pre-care experiences made them feel uncomfortable or because they did not think it would be helpful. This sense of discomfort when talking about previous experiences often appeared to be associated with a lack of trust in adults and services. Participants also discussed feeling embarrassed, uncomfortable with the amount of attention placed on them, and unsure of how to act when describing events.

I don't really like talking about my past too much, it's, it's like all attentions on me and it's like awkward.

It don't change nothing, it don't mean it go back. It still happened you can't make it [go away]

From those who identified talking as an unhelpful strategy, many held the attitude that, as events are in the past, talking about events will not change anything. Some also felt that it acted as a barrier to moving forward. This was particularly reported by young people who

For peer review only - http://bmjopen.bmj.com/site/about/guidelines.xhtml

were also reporting high mental health difficulties, including current distressing intrusive memories of their maltreatment.

I'm not saying "suck it up and be a man" but just try and just keep your mind off it, keep doing what you're doing cos you don't really want to let the past like take over you. Because the past is in the past and I really don't want to bring my past up. I'm trying to move forward for the minute but obviously if people are bringing it up, I can't.

In general, many participants endorsed avoidance of maltreatment memories and of talking about their past as a primary way to cope. In most cases this involved distracting themselves with activities, such as watching TV and spending time with friends. Indeed in many cases when talking about their support systems, young people endorsed that adults helping to distract them was helpful (e.g changing the topic; social workers taking them to lunch; see Theme 2 quotes). These distractions shifted the focus of their attention onto something else, allowing them to avoid thinking about their early experiences.

[in response to question about coping] *just watch tv or something.*

I would go on the PlayStation and just kill people.

In the case of adolescents who were struggling with mental health difficulties, there were more frequent examples of destructive coping strategies, particularly self-harm and drug and alcohol use. Many of these young people reported that they did not have any other way of coping with their emotional difficulties.

I pretend to be okay when I'm not and let emotions build up until eventually I just break. Getting drunk a lot, doing a lot of drugs.

I cry and I hurt myself that's literally the only way I know how to deal with things anymore.

Discussion

For peer review only - http://bmjopen.bmj.com/site/about/guidelines.xhtml

This study aimed to learn more about the views of young people in out-of-home care, in relation to coping, support and help-seeking with emotional difficulties. Across the interviews there were core common themes, including the importance of having a consistent and trusted adult with whom they could share worries. Carers were identified as the primary source of emotional support by most young people. While many participants talked about the importance of talking to your support system as a means of coping, most also thought that it was not helpful to talk about their pre-care experiences. Adolescents experiencing mental health difficulties also commonly struggled to identify adaptive coping strategies or support systems.

Similar to other qualitative work with young people in care [9,12], participants viewed the support of a trusted adult as key to coping with daily stress and interpersonal difficulties, and with more ongoing mental health difficulties. In the vast majority of cases, the carer (or a key worker if in residential care) was viewed as the primary source of this support, although some young people talked about this support also coming from their social worker, a trusted staff member at school, or friend. Trustworthiness was discussed as particularly central to the young person feeling supported, including the perception that the adult would be able to keep information confidential. A lack of trust was often expressed as a key concern and potential barrier to seeking help. While some young people had positive relationships with their social workers, many participants discussed changes in social workers as another key barrier to building a trusted relationship and seeking support for emotional difficulties, reflecting similar findings by Wood and Selwyn in their qualitative work, where young people in care also discussed the challenges of navigating regular changes in social workers and the ultimate negative impact on their subjective wellbeing [25]. Findings highlights the need for services to explore policies around mitigating some of the consequences of social worker changes on the young person's wellbeing. Social work has

For peer review only - http://bmjopen.bmj.com/site/about/guidelines.xhtml

some of the highest burn-out rates of any profession. Providing more robust training and support to social workers is considered key to reducing social work changes [26]. However, given some change is inevitable, policies for how these changes happen are crucial, to minimise the potential eroding of trust.

While many participants talked about going to their carer for support if they were feeling worried or upset (e.g., if they were upset about something at school), many at the same time emphatically stated that they did not think it was useful to talk about their maltreatment experiences, with either their carer or mental health professionals. While this was the case across much of the sample, it is perhaps most concerning for those young people who discussed experiencing significant mental health difficulties. This sub-set of the sample most commonly reported feeling that they had no person they could turn to for support, that they did not want to talk to a professional about their maltreatment, and had no strategies for coping with their mental health beyond self-harm and drug and alcohol abuse. Beliefs around not talking about maltreatment or trauma present a particular challenge to mental health and social-care professionals. While it is understandable that young people may not want to talk about distressing early experiences, avoiding talking or thinking about traumatic experiences (i.e., thought suppression) is a key maintainer of poor mental health outcomes [15], including for young people in care [16]. Further, a key component of evidenced-based trauma-focused psychological support involves processing the traumatic experiences, commonly via a trauma narrative [e.g., 27]. There remains an urgent need to understand how services can best engage young people in care in mental health treatments that might involve talking about their experiences, particularly for a group where developing trust and a strong therapeutic relationship is essential [10].

This may be partly achieved by having systems, including at a social-care and education level, where staff are appropriately trained and supported in engaging with these

For peer review only - http://bmjopen.bmj.com/site/about/guidelines.xhtml

challenging conversations with young people from earlier on in their time in care, so young people know that it is safe to talk about these experiences if they want to. Many services in the UK would report using "trauma-informed" approaches to their practice [28], which would include creating safe spaces for young people to talk about their experiences. However, the evidence-base for how these approaches are genuinely implemented and their effectiveness, remains scarce. Relatedly, many young people reported feeling stigmatised by being in care, including by professionals (e.g., teachers) and peers – a finding that is also in line with other qualitative work with this group [e.g., 25]. It will therefore also be important for services to consider how to address issues of stigma, which can be a barrier to help-seeking. Strength and relationship-based approaches may be useful here [29], but remain poorly researched in terms of how they are used in practice or their general effectiveness. These remain important areas for future research, to focus not just on targeting psychopathology but more broadly improving the emotional wellbeing of these young people.

While only the minority of participants in this study had received professional mental health support, including via CAMHS and school counselling, views on this support were also mixed. In their 2008 review of qualitative literature on children in care and mental health services, Davies and Wright highlighted that many young people felt wary and unsure of professional mental health support, and highlighted the importance of developing trust [9]. Ten years later, the current study found that young people in care are still experiencing these same concerns. There remains an urgent need to improve understanding of the service-level barriers to accessing professional support, including access to evidence-based psychological interventions (e.g., see review [30]). Within the current UK CAMHS system, thresholds for professional mental health support often mean young people are in crisis, with complex mental health needs, by the time they can access support (e.g., see [31]). Exploring feasible

For peer review only - http://bmjopen.bmj.com/site/about/guidelines.xhtml

avenues for providing effective evidence-based support, before reaching crisis, is clearly an important avenue for addressing the mental health needs of these young people.

Limitations

While this study has key strengths, including a focus on an under-researched group, and the use of qualitative interviews to provide an in-depth exploration of their views on coping and wellbeing, findings should also be considered in light of key limitations. First, we used opportunity sampling methods. All young people were participants in a larger quantitative study of mental health and wellbeing. Young people who did not want to talk about their experiences and mental health would likely have declined participating in this study. Participants also all came from two local authorites in England. While findings cannot necessarily be generalised to other young people in out-of-home care, it is worth noting the significant overlap between these findings and qualitative work with young people in care and foster carers in the UK, US, and Australia [e.g., 9,19]. Finally, quantitative research is needed to provide more robust evidence on the consequences of different support systems and coping strategies in relation to the mental health of these young people.

Conclusions

In sum, while there were positives to being in care and many young people felt well supported by their carer, results of this qualitative study identified key areas to be addressed to improve support for the emotional wellbeing of this group. This included improving the consistency of social workers and carers to allow young people to develop trusted relationships with key adults, targeting broader concerns around stigma and trust that may act as a barrier to help-seeking, and ensuring young people are receiving appropriate evidence-based psychological support in a more timely manner, before reaching crisis point. Addressing these issues remains a significant challenge for services, but failing to provide

For peer review only - http://bmjopen.bmj.com/site/about/guidelines.xhtml

adequate support, both in terms of informal day-to-day support and professional services, can have a substantial impact on the trajectory of these young people.

For peer review only

For peer review only - http://bmjopen.bmj.com/site/about/guidelines.xhtml

Contributors: RMH is the lead researcher on the project and PI on the ESRC grant. She led the conceptualisation of the project, conducted a proportion of the interviews, and was responsible for overall project management. SLH was involved in the conceptualisation of the project, including the design of the interview schedule and also contributed to discussion on the themes and feedback on the manuscript. RM-S was involved in the conceptualisation of the project and contributed to the discussion about the themes and feedback on the manuscript. EE was the research assistant on the project and conducted a proportion of the interviews and contributed to transcribing, data analysis, and feedback on the manuscript. ER-E was involved in the transcribing, contributed to data analysis and provided feedback on the manuscript. TH contributed to transcribing and feedback on the codes and themes. The authors also wish to sincerely thank the many social-care and mental health service staff, carers, and young people who contributed to the design of this project and from whom we continue to learn so much.

Funding: This research was funded by an ESRC Future Leader Grant (ES/N01782X/1) and University of Bath Prize Fellowship awarded to RMH.

Competing Interests: None to declare.

Data sharing: Anonymised transcripts will be available on request on a case-by-case basis.

For peer review only - http://bmjopen.bmj.com/site/about/guidelines.xhtml

References

[1] Department for Education. Statistics: looked after children. London: Author. 2018.

[2] Pecora PJ, White CR, Jackson LJ, Wiggins T. Mental health of current and former recipients of foster care: A review of recent studies in the USA. Child & Family Social Work. 2009; 14(2):132-146.

[3] Hiller RM, St Clair MCS. The emotional and behavioural symptom trajectories of children in long-term out-of-home care in an English local authority. Child Abuse & Neglect. 2018; 81:106-117.

[4] Newton RR, Litrownik AJ, Landsverk JA. Children and youth in foster care: Disentangling the relationship between problem behaviors and number of placements. Child Abuse & Neglect. 2000; 24(10):1363-1374.

[5] Ford T, Vostanis P, Meltzer H, Goodman R. Psychiatric disorder among British children looked after by local authorities: comparison with children living in private households. The British Journal of Psychiatry. 2007; 190(4):319-325.

[6] McMillen JC, Zima BT, Scott Jr LD, Auslander WF, Munson MR, Tollie MT, Spitznagel EL. Prevalence of psychiatric disorders among older youths in the foster care system. Journal of the American Academy of Child & Adolescent Psychiatry. 2005; 44(1):88-95.

[7] Oswald SH, Heil K, Goldbeck L. History of maltreatment and mental health problems in foster children: A review of the literature. Journal of Pediatric Psychology. 2009; 35(5):462-472.

[8] Jones R, Everson-Hock ES, Papaioannou D, Guillaume L, Goyder E, Chilcott J, ... & Swann C. Factors associated with outcomes for looked-after children and young people: a correlates review of the literature. Child: Care, Health and Development. 2011; 37(5):613-622.

For peer review only - http://bmjopen.bmj.com/site/about/guidelines.xhtml

[9] Davies J, Wright J. Children's voices: A review of the literature pertinent to looked-after children's views of mental health services. Child and Adolescent Mental Health. 2008; 13(1):26-31.

[10] Shaw M, De Jong M. Child abuse and neglect: a major public health issue and the role of child and adolescent mental health services. The Psychiatrist. 2012; 36(9):321-325.

[11] Ahmed K, Windsor L, Scott S. In their own words: abused children's perceptions of care provided by their birth parents and foster carers. Adoption & Fostering. 2015; 39(1): 21-37.

[12] Sinclair I, Wilson K. Matches and mismatches: The contribution of carers and children to the success of foster placements. British Journal of Social Work. 2003; 33(7):871-884.

[13] Rock S, Michelson D, Thomson S, Day C. Understanding foster placement instability for looked after children: A systematic review and narrative synthesis of quantitative and qualitative evidence. British Journal of Social Work. 2013; 45(1):177-203.

[14] Murray L, Creswell C, Cooper PJ. The development of anxiety disorders in childhood: an integrative review. Psychological Medicine. 2009; 39(9):1413-1423.

[15] Trickey D, Siddaway AP, Meiser-Stedman R, Serpell L, Field AP. A meta-analysis of risk factors for post-traumatic stress disorder in children and adolescents. Clinical Psychology Review. 2012; 32(2):122-138.

[16] Hiller RM, Meiser-Stedman R, Elliott E, Banting R, Halligan SL. Cognitive predictors of (complex) posttraumatic stress: A longitudinal study of young people in out-of-home care. Journal of Child Psychology and Psychiatry. 2020; In Press.

[17] Marshall B, Cardon P, Poddar A, Fontenot R. Does sample size matter in qualitative research?: A review of qualitative interviews in IS research. Journal of computer information systems. 2013; 54(1):11-22.

[18] Vasileiou K, Barnett J, Thorpe S, Young T. Characterising and justifying

For peer review only - http://bmjopen.bmj.com/site/about/guidelines.xhtml

sample size sufficiency in interview-based studies: systematic analysis of qualitative health research over a 15-year period. BMC Medical Research Methodology. 2018; 18(1):148.

[19] Ahrens KR, DuBois DL, Garrison M, Spencer R, Richardson LP, Lozano P. Qualitative exploration of relationships with important non-parental adults in the lives of youth in foster care. Children and Youth Services Review. 2011; 33(6):1012-1023.

[20] Johnson E, Menna R. Help seeking among adolescents in foster care: A qualitative study. Children and Youth Services Review. 2017; 76:92-99.

[21] Docherty S, Sandelowski M. Focus on qualitative methods: Interviewing children. Research in Nursing & Health. 1999; 22(2):177-185.

[22] Van Wesel F, Boeije H, Alisic E, Drost S. I'll be working my way back: A qualitative synthesis on the trauma experience of children. Psychological Trauma: Theory, Research, Practice, and Policy. 2012; 4(5):516.

[23] Braun V, Clarke V. Using thematic analysis in psychology. Qualitative Research in Psychology. 2006; 3(2):77-101.

[24] Braun V, Clarke V, Hayfield N, Terry G. Thematic analysis. In P Liamputtong. Handbook of Research Methods in Health Social Sciences. Singapore: Springer. 2019. 843-60.

[25] Wood M, Selwyn J. Looked after children and young people's views on what matters to their subjective well-being. Adoption & Fostering. 2017; 41(1):20-34.

[26] Kim H, Ji J, Kao D. Burnout and physical health among social workers: A three-year longitudinal study. Social Work. 2011; 56(3):258-268.

[27] Deblinger E, Mannarino AP, Cohen JA, Runyon MK, Steer RA. Trauma-focused cognitive behavioral therapy for children: impact of the trauma narrative and treatment length. Depression and Anxiety. 2011; 28(1):67-75.

For peer review only - http://bmjopen.bmj.com/site/about/guidelines.xhtml

[28] Beyerlein BA, Bloch E. Need for trauma-informed care within the foster care system: A policy issue. Child Welfare. 2014; 93(3):7.

[29] Brooks R, Brooks S. Creating resilient mindsets in children and adolescents: A strength-based approach for clinical and nonclinical populations. In Resilience interventions for youth in diverse populations. Springer, New York, NY. 2014; 59-82.

[30] Leve LD, Harold GT, Chamberlain P, Landsverk JA, Fisher PA, Vostanis P. Practitioner review: children in foster care–vulnerabilities and evidence-based interventions that promote resilience processes. Journal of Child Psychology and Psychiatry. 2012; 53(12): 1197-1211.

[31] Blow E. Children and young people's mental health – policy, CAMHS services, funding and education. London: House of Commons Library. 2015.

For peer review only

For peer review only - http://bmjopen.bmj.com/site/about/guidelines.xhtml

Table 1. *Sample demographics.*

**Young person descriptives (N = 25)**	
Age, *M* (*SD*), Range	13.0 (2.24)
Sex (% female)	56%
Ethnicity (% Caucasian)	80%
Age entered care, *M* (*SD*)	8.7 yrs (4.2)
Time in care, *M* (*SD*)	4.2 yrs (3.6)
% who had multiple placements	80%
Type of placement	
Non-biological foster carer	76%
Biological foster carer (e.g., grandmother)	16%
Residential care home	8%

For peer review only

For peer review only - http://bmjopen.bmj.com/site/about/guidelines.xhtml

Table 2. *Themes and subthemes from thematic analysis.*

Themes	Sub-themes
**Being in care could create a sense of safety but some young people struggled with a lack of control and agency**	Being in care can create a sense of safety and of being part of a family.
	Negative aspects of being in care included feeling different and not being listened to or respected.
**Social support was considered key to coping but not always available, while access to professional support was inconsistent.**	Day-to-day support from a trusted adult was identified as very important for wellbeing.
	Formal mental health support was often unavailable and opinions on usefulness were mixed.
**Talking about problems was seen as important, but maladaptive coping strategies were also reported**	Talking about worries or challenges was identified as a helpful strategy for coping with negative emotions.
	A majority avoided talking about their pre-care experiences and in the context of mental health difficulties maladaptive coping was common.

For peer review only

For peer review only - http://bmjopen.bmj.com/site/about/guidelines.xhtml

Child qualitative interview

Thank you for talking to me today. Like it said in the letter I'm interested in finding out a bit more about your views on how you cope and where you might go for support or help if you're struggling with thoughts or feelings. Remember, you don't need to talk about your experiences in your home. You can if you want to but we won't be asking you to. What I'm really interested in is what happened *after* this, when you came in to care. This isn't like a test at school, there are no right or wrong answers, it's just about talking honestly and sharing your opinion. If you want to stop at any time or take a break just let me know. Does that sound ok?

Demographics

- Do you remember how old you were when you first came in to care?
- How long have you lived in [current placement]?

Reactions

- How have you felt since you came in to care?
  - Are there good parts?
  - What are some of the harder parts?

Support

- If you're having a hard time, is there anything you do to try and make yourself feel better or different?
  - (*example might be argument with friend at school; through to mental health difficulty if yp has disclosed this*)

- Is there anyone you talk to, to try to make yourself feel better?
  - Who? Is that helpful/unhelpful? Why?
  - Anyone else?
  - Have you ever talked to a professional? Like a psychologist or school counsellor? What was this like? What was helpful/unhelpful?

- Do you ever talk to anyone about what you experienced before you came into care?
  - Why/why not?
  - What helps/doesn't help?

Support wishes

- What support do you wish you had got that you didn't?
  - (example might be – *if you were asked to create the best possible care system to support young people who were struggling with their thoughts and feelings, what would it look like?*)
- If you had a friend that went through the same things as you, how would you help them? What advice might you give them to help them cope with their thoughts and feelings?

Is there anything else you want to add that we haven't covered?

For peer review only - http://bmjopen.bmj.com/site/about/guidelines.xhtml

Reporting checklist for qualitative study.

Based on the SRQR guidelines.

Instructions to authors

Complete this checklist by entering the page numbers from your manuscript where readers will find each of the items listed below.

Your article may not currently address all the items on the checklist. Please modify your text to include the missing information. If you are certain that an item does not apply, please write "n/a" and provide a short explanation.

Upload your completed checklist as an extra file when you submit to a journal.

In your methods section, say that you used the SRQRreporting guidelines, and cite them as:

O'Brien BC, Harris IB, Beckman TJ, Reed DA, Cook DA. Standards for reporting qualitative research: a synthesis of recommendations. Acad Med. 2014;89(9):1245-1251.

		Reporting Item	Page Number
**Title**			
	#1	Concise description of the nature and topic of the study identifying the study as qualitative or indicating the approach (e.g. ethnography, grounded theory) or data collection methods (e.g. interview, focus group) is recommended	1

For peer review only

Abstract

	#2	Summary of the key elements of the study using the abstract format of the intended publication; typically includes background, purpose, methods, results and conclusions	2

Introduction

Problem formulation	#3	Description and signifcance of the problem / phenomenon studied: review of relevant theory and empirical work; problem statement	4-5
Purpose or research question	#4	Purpose of the study and specific objectives or questions	5

Methods

Qualitative approach and research paradigm	#5	Qualitative approach (e.g. ethnography, grounded theory, case study, phenomenolgy, narrative research) and guiding theory if appropriate; identifying the research paradigm (e.g. postpositivist, constructivist / interpretivist) is also recommended; rationale. The rationale should briefly discuss the justification for choosing that theory, approach, method or technique rather than other options available; the assumptions and limitations implicit in those choices and how those choices influence study conclusions and transferability.	7-8

For peer review only

		As appropriate the rationale for several items might be discussed together.	
Researcher characteristics and reflexivity	#6	Researchers' characteristics that may influence the research, including personal attributes, qualifications / experience, relationship with participants, assumptions and / or presuppositions; potential or actual interaction between researchers' characteristics and the research questions, approach, methods, results and / or transferability	7
Context	#7	Setting / site and salient contextual factors; rationale	6
Sampling strategy	#8	How and why research participants, documents, or events were selected; criteria for deciding when no further sampling was necessary (e.g. sampling saturation); rationale	6
Ethical issues pertaining to human subjects	#9	Documentation of approval by an appropriate ethics review board and participant consent, or explanation for lack thereof; other confidentiality and data security issues	6
Data collection methods	#10	Types of data collected; details of data collection procedures including (as appropriate) start and stop dates of data collection and analysis, iterative process, triangulation of sources / methods, and modification of procedures in response to evolving study findings; rationale	6

For peer review only

Data collection instruments and technologies	#11	Description of instruments (e.g. interview guides, questionnaires) and devices (e.g. audio recorders) used for data collection; if / how the instruments(s) changed over the course of the study	6-8
Units of study	#12	Number and relevant characteristics of participants, documents, or events included in the study; level of participation (could be reported in results)	6-8
Data processing	#13	Methods for processing data prior to and during analysis, including transcription, data entry, data management and security, verification of data integrity, data coding, and anonymisation / deidentification of excerpts	6-8
Data analysis	#14	Process by which inferences, themes, etc. were identified and developed, including the researchers involved in data analysis; usually references a specific paradigm or approach; rationale	6-8
Techniques to enhance trustworthiness	#15	Techniques to enhance trustworthiness and credibility of data analysis (e.g. member checking, audit trail, triangulation); rationale	7-8
**Results/findings**			
Syntheses and interpretation	#16	Main findings (e.g. interpretations, inferences, and themes); might include development of a theory or model, or integration with prior research or theory	8-16

For peer review only

Links to empirical data	#17	Evidence (e.g. quotes, field notes, text excerpts, photographs) to substantiate analytic findings	8-16
**Discussion**			
Intergration with prior work, implications, transferability and contribution(s) to the field	#18	Short summary of main findings; explanation of how findings and conclusions connect to, support, elaborate on, or challenge conclusions of earlier scholarship; discussion of scope of application / generalizability; identification of unique contributions(s) to scholarship in a discipline or field	16
Limitations	#19	Trustworthiness and limitations of findings	19
**Other**			
Conflicts of interest	#20	Potential sources of influence of perceived influence on study conduct and conclusions; how these were managed	21
Funding	#21	Sources of funding and other support; role of funders in data collection, interpretation and reporting	21

Notes:

- 21: n/a (blinded) The SRQR checklist is distributed with permission of Wolters Kluwer © 2014 by the Association of American Medical Colleges. This checklist was completed on 30. July 2019 using https://www.goodreports.org/, a tool made by the EQUATOR Network in collaboration with Penelope.ai

For peer review only

BMJ Open

Coping and support-seeking in out-of-home care: A qualitative study of the views of young people in care in England

Journal:	*BMJ Open*
Manuscript ID	bmjopen-2020-038461.R2
Article Type:	Original research
Date Submitted by the Author:	22-Oct-2020
Complete List of Authors:	Hiller, Rachel; University of Bath, Department of Psychology<br>Halligan, Sarah; University of Bath, Psychology; University of Cape Town, Department of Psychiatry and Mental Health<br>Meiser-Stedman , Richard ; University of East Anglia,<br>Elliott, Elizabeth; University of Bath, Department of Psychology; University of Reading, School of Psychology and Clinical Language Science<br>Rutter-Eley, Emily; University of Reading, School of Psychology and Clinical Language Science<br>Hutt, Tilly; University of Bath, Department of Psychology
**Primary Subject Heading**:	Mental health
Secondary Subject Heading:	Qualitative research, Paediatrics
Keywords:	Child protection < PAEDIATRICS, Child & adolescent psychiatry < PSYCHIATRY, Quality in health care < HEALTH SERVICES ADMINISTRATION & MANAGEMENT

SCHOLARONE™
Manuscripts

I, the Submitting Author has the right to grant and does grant on behalf of all authors of the Work (as defined in the below author licence), an exclusive licence and/or a non-exclusive licence for contributions from authors who are: i) UK Crown employees; ii) where BMJ has agreed a CC-BY licence shall apply, and/or iii) in accordance with the terms applicable for US Federal Government officers or employees acting as part of their official duties; on a worldwide, perpetual, irrevocable, royalty-free basis to BMJ Publishing Group Ltd ("BMJ") its licensees and where the relevant Journal is co-owned by BMJ to the co-owners of the Journal, to publish the Work in this journal and any other BMJ products and to exploit all rights, as set out in our licence.

The Submitting Author accepts and understands that any supply made under these terms is made by BMJ to the Submitting Author unless you are acting as an employee on behalf of your employer or a postgraduate student of an affiliated institution which is paying any applicable article publishing charge ("APC") for Open Access articles. Where the Submitting Author wishes to make the Work available on an Open Access basis (and intends to pay the relevant APC), the terms of reuse of such Open Access shall be governed by a Creative Commons licence – details of these licences and which Creative Commons licence will apply to this Work are set out in our licence referred to above.

Other than as permitted in any relevant BMJ Author's Self Archiving Policies, I confirm this Work has not been accepted for publication elsewhere, is not being considered for publication elsewhere and does not duplicate material already published. I confirm all authors consent to publication of this Work and authorise the granting of this licence.

For peer review only

Coping and support-seeking in out-of-home care: A qualitative study of the views of young people in care in England

Rachel M Hiller[1], Sarah L Halligan[1,2], Richard Meiser-Stedman[3], Elizabeth Elliott[1,4], Emily Rutter-Eley[4], Tilly Hutt[1]

[1] Department of Psychology, University of Bath, UK

[2] Department of Psychiatry and Mental Health, University of Cape Town, South Africa

[3] Department of Clinical Psychology, Norwich Medical School, University of East Anglia, UK

[4] School of Psychology and Clinical Language Science, University of Reading, UK

* Corresponding Author:

Dr Rachel Hiller

Department of Psychology

University of Bath

P. 01225 383794

E. R.Hiller@bath.ac.uk

Main text word count: 5, 755

For peer review only - http://bmjopen.bmj.com/site/about/guidelines.xhtml

Abstract

Objectives: Young people who have been removed from their family home and placed in out-of-home care have commonly experienced abuse, neglect and/or other forms of early adversity. High rates of mental health difficulties have been well-documented in this group. The aim of this research was to explore the experiences of these young people within the care system, particularly in relation to support-seeking and coping with emotional needs, to better understand feasible and acceptable ways to improve outcomes for this group.

Design and Study-Setting: This study used 1:1 semi-structured qualitative interviews with young people in out-of-home care in England, to provide an in-depth understanding of their views of coping and support for their emotional needs, both in terms of support networks and experiences with mental health services. Participants were 25 10-16 years olds (56% female) and included young people living with non-biological foster carers, kinship carers, and in residential group homes.

Results: Participants described positive (e.g. feeling safe) and negative (e.g. feeling judged) aspects to being in care. Carers were identified as the primary source of support, with a supportive adult central to coping. Views on support and coping differed for young people who were experiencing more significant mental health difficulties, with this group largely reporting feeling unsupported and many engaging in self-harm. The minority of participants had accessed formal mental health support, and opinions on usefulness were mixed.

Conclusions: Results provide insight, from the perspective of care-experienced young people, about both barriers and facilitators to help-seeking, as well as avenues for improving support.

Key words: child maltreatment; care-experienced; adolescents; foster care; emotional wellbeing; mental health; help-seeking; qualitative

For peer review only - http://bmjopen.bmj.com/site/about/guidelines.xhtml

Strengths and Limitations of the Study:

- This study is focused on young people living in out-of-home care, representing an under-researched group where there remains significant need.
- The study recruited from two English local authorities.
- Using 1:1 qualitative interview methods we gained an in-depth understanding of the views and experiences of these young people, in terms of coping and help-seeking around mental health.
- As this study uses qualitative methods, we cannot conclude whether different types of reported coping might be associated with better or worse outcomes.

For peer review only

For peer review only - http://bmjopen.bmj.com/site/about/guidelines.xhtml

Introduction

The most common reason a child would be removed from their home and placed in out-of-home care is due to the experience of abuse and/or neglect [e.g., 1,2]. In the UK, it is also the case that most young people who enter care are removed from their home when school-aged or older, meaning early experiences of maltreatment have often been ongoing for a number of years [1]. Once in care many continue to face ongoing instability. Separation from siblings is common, as are moves between different placement providers, resulting in the potential for inconsistent support [3-4]. Given these accumulative experiences, it is perhaps unsurprising that there is well-established evidence that young people in out-of-home care experience high rates of psychological difficulties compared to their peers [5-7].

Failing to adequately address the psychological or emotional needs of young people in care has been identified as a key driver of many of the well-documented poor outcomes that can categorise this group, including school attainment and later rates of homelessness and unemployment [8]. Yet, despite the long-documented poor outcomes, there has been limited evidence of improvements in the overall picture of mental health for children in care in the UK over the past decade [1]. One concern is that these young people can fall between the gaps of social-care and national health service provisions, which can result in poor access to mental health services [9-10]. With resource issues affecting child and adolescent mental health services (CAMHS) in many countries, it is also largely impossible for these services to address the large number of young people in care who may benefit from professional mental health support.

In the face of potentially limited professional support services, young people will inevitably develop day-to-day coping strategies and support networks – whether perceived to be adaptive or maladaptive. Some qualitative work has investigated the views of young people in care in relation to support networks, and specifically their relationships with carers,

For peer review only - http://bmjopen.bmj.com/site/about/guidelines.xhtml

finding developing trust, consistent boundaries, and empathy were viewed as important [11-12]. However, there is less evidence for how they may or may not engage with other support networks, including peers, school, and social workers. Such evidence is potentially particularly important for this group, given one-third of young people in care in the UK will have more than one carer in any given year [1], and given the well-documented cyclical association between placement breakdowns and emotional difficulties [4,13]. Beyond the role of external support networks, we know little about what strategies young people in care may be using themselves to manage their emotional wellbeing. While many young people in care struggle with emotional difficulties, it is important to note that not all do. Evidence from the wider child mental health field shows that engaging in certain types of coping strategies can be associated with better or worse mental health difficulties [14-15], including in care-experienced young people [16]. Understanding how young people in care cope with emotional difficulties, particularly in the face of potentially disrupted support networks, is crucial to understanding both the experiences of this group, and the avenues for improving formal and informal support.

Using a semi-structured qualitative approach we sought to explore the following questions: (1) where do young people in care seek support for emotional difficulties, both in terms of social support and professional services?; (2) what do they view as barriers to seeking help?; and (3) what coping strategies do they use when experiencing emotional difficulties? This study included a focus on more day-to-day coping and help-seeking (e.g., if they felt stressed) and coping and help-seeking around more significant mental health difficulties.

Method

Recruitment and Sample

For peer review only - http://bmjopen.bmj.com/site/about/guidelines.xhtml

Ethical approval was granted from the University of Bath Psychology- and Social Care- research ethics committees, along with permission from the participating local authorities. Using opportunity sampling, we recruited 25 young people aged 10 – 16 years old. Participants were all part of a larger quantitative study on the mental health and wellbeing of young people in care [16]. The sample size is based on general guidance for qualitative methodology [see 17-18]. While clear guidance for sample sizes in qualitative research remains limited, our sample size was chosen to be in line with, or larger than, similar studies in the field [e.g., 19-20]. It was also based on our previous qualitative work with young people in terms of the sample required to reach saturation, with capacity to continue recruitment if it was felt saturation was not reached or the data was inadequate (which was ultimately not the case). The sample size also allowed us to explore views of older and younger children within our age range, and include young people with a range of mental health needs, from those who were managing well to those struggling with chronic mental health difficulties.

Participants came from two local authorities in England. One was a smaller-sized local authority and one was medium-sized, with both in largely urban areas. Informed consent for the young person's participation was provided by their social worker, while the young person provided informed assent (or consent if 16 years old). Eligibility for participation in the quantitative study were: aged 10-17 years old, on full care order if under 16 years old (where the local authority has acquired parental responsibility for the young person), absence of developmental disability that would preclude the child from mainstream schooling, and absence of significant current risk (e.g., current severe suicidality). Young people could be in any style of placement, including foster placement with a non-biological carer, kinship placement, residential care home. Descriptive information on the sample is presented in Table 1.

For peer review only

Procedure and Measures

In-person 1:1 interviews were conducted by two researchers. The lead researcher (RH) had prior training and experience in qualitative research, while the other (EE) was experienced researching with vulnerable young people and adults and received further training specifically on qualitative procedures. We used a 1:1 semi-structured interview format, rather than focus group, due to the potential for sensitive information being discussed that young people might not have wished to disclose in a group. For one of these interviews two siblings requested to complete the interview together, while for two interviews the carer remained present at the request of the young people. All others (n = 22) had only the young person and interviewer present. RH and EE met regularly throughout the study to monitor the quality of the interviews and reflect on the content. All interviews were audio recorded and later transcribed verbatim. As a thank you for volunteering their time, the young people each received a £10 voucher. As per standard risk management and safeguarding procedures, and where appropriate, summary letters were provided to social workers to support potential referrals to mental health services.

Descriptives and semi-structured interview. Basic descriptive information was collected from the young person's social worker, as part of the larger quantitative study. This included the age and reason that the young people had entered care. The semi-structured interview was developed based on the literature on qualitative interviewing techniques and qualitative and quantitative work in the child mental health field [e.g., 9, 21-22]. Interviews broadly covered four core areas: (i) views on what is broadly helpful and unhelpful within the care system, (ii) coping and support for children's mental health and wellbeing (including at home, within social-care, and experiences around mental health services), (iii) coping and support for trauma-related mental health more specifically (e.g., how young people cope with memories of their maltreatment), and (iv) views on ideal support. Questions were primarily

For peer review only - http://bmjopen.bmj.com/site/about/guidelines.xhtml

focused on the young person's own experiences (see Appendix 1 for interview schedule). Additionally, participants were also asked what advice they might give a friend in a similar situation, to elicit broader information from young people who might have struggled to answer these questions about themselves. Questions were primarily open-ended (e.g., "If you're having a hard time what do you do to make yourself feel better or different?"). Some direct prompting and questioning were used to clarify comments and gain further insight (e.g., "Was that helpful?"). Interviews lasted from 6-32 minutes (M = 15.36 minutes).

Data Analyses

Transcripts were analysed using a reflexive thematic analysis approach [23-24] in NVivo 12. Given the exploratory nature of the study, thematic analysis was chosen as the data analytic approach as it allows for a detailed exploration of the data and does not rely on a specific theoretical framework. Co-author (ER), who was not involved in any of the interviews or the broader quantitative study, coded all transcripts. While the purpose of qualitative research is not to completely remove or minimise the influence of researcher subjectivity, a coder who was had not been involved in study design or data collection was used, to reduce potential biases. She first read all transcripts to gain an understanding of the overall data (data immersion) and then systematically coded each transcript using an iterative process [24]. Following the systematic coding of all transcripts, codes were grouped to form overarching themes. The broader themes were also separated into subthemes, to accurately capture more fine grain information provided by participants and ensure the themes appropriately reflected different viewpoints. Co-authors (TH) independently explored all (un-coded) transcripts and generated their own key themes. A consensus meeting showed strong agreement between the primary themes. There was not capacity within the study to seek further input from participants at this point, however reflective practices were used throughout all interviews to ensure clear understanding of the discussion and to minimise any

For peer review only - http://bmjopen.bmj.com/site/about/guidelines.xhtml

chance of misinterpreting what was being said (e.g., reflecting, summarising back to participants and seeking further clarification where needed).

Patient and Public Involvement

This study occurred as part of a series of projects, which involves extensive consultation with services, as well as feedback from care-experienced young people and foster carers. This feedback particularly shaped the research questions and use of qualitative methods. Young people and carers were not involved in recruitment for the project. There has been wide written and verbal feedback to stakeholders as part of the larger project.

Results

Descriptive Information

The sample comprised of 14 females and 11 males, aged 10 to 16 years old. They had entered care between infancy and 16 years old, and had been in care from 6 months to almost 14 years, while 80% of the sample had had more than one placement since entering care. Most (76%) were living in a foster placement with a non-biological carer, while four were in a kinship placement (e.g., with a grandmother), and two lived in residential care homes. All were in care due to the experience of abuse and/or neglect.

Interview Themes

Thematic analysis identified three primary themes covering the broader experience of being in care (Theme 1); the centrality of social support to wellbeing and mixed views on professional help (Theme 2); and the use of both adaptive and maladaptive day-to-day coping strategies (Theme 3). A list of themes and subthemes can be found in Table 2.

Theme 1: Being in care could create a sense of safety but some young people struggled with a lack of control and agency

When asked what it was like to be in care, most, but not all participants expressed that being in care was a better or safer alternative to living with their biological family and that

For peer review only

they felt safer in care. However, almost all discussed occasions of feeling stigmatised, as well as experiences of being poorly supported by services.

Subtheme 1: Being in care can create a sense of safety and of being part of a family. The majority of participants reported that feeling safe and secure was a particularly positive aspect of being in care. Some participants also described experiencing a sense of relief when first coming into care, recognising that it was not appropriate for them to remain in an unsafe environment.

Relieved actually from going to a bad environment into somewhere I thought could be quite safe. [16yo]

To know at the end of the day you can go home, instead of thinking 'OK, I'm going to go home tonight, am I going to find my mum crying on the floor again drunk?' [12yo]

The majority of young people discussed how they have felt "normal" and like part of a family since coming into care. This was expressed regardless of participants' current relationships with biological family, with many describing strong and important bonds with both their biological and foster family. Feeling like part of a family was especially valued and some expressed their appreciation for the support and sense of belonging provided by their foster carers.

Just feel like an ordinary family really, like an ordinary person. Sometimes I don't even realise I'm in care. [11yo]

I'm lucky I ended up here. I'm so goddamn lucky I ended up here, coz this is my home now, it's my family and I love 'em, I love 'em, I actually do, coz they're my family. [12yo]

In some cases, these positive aspects were reported to facilitate improvements within different areas of participants' lives, such as at school and when coping with difficult situations and emotions.

For peer review only - http://bmjopen.bmj.com/site/about/guidelines.xhtml

They [carers] *like understand what I'm saying and like if I'm annoyed they help me to know how to calm down.* [10yo]

I started knuckling down at school, because I felt safe. [12yo]

Subtheme 2: Negative aspects of being in care included feeling different and not being listened to or respected. Most participants discussed feeling "different" because they were a young person in care, and were concerned about others' judgement. Mandatory forms and compulsory meetings with social workers could create difficult situations for participants, or *"annoying"* disruptions, which served as regular reminders that they were different from other children. Alongside this, concerns about others' (e.g., teachers, peers) lack of understanding meant that many participants were very conscious of being judged or stigmatised, particularly within the school context.

Most people think you are naughty 'cause you're in care. [10yo]

Like the social workers coming in here [school] or whatever… it's awkward 'cause like a normal person wouldn't really have that. [10yo]

Amongst adolescents and particularly those struggling with mental health difficulties, feeling different from their peers was often accompanied by complaints of lacking freedom and control, generating a feeling of restricted independence. This led some older participants to express their desire to leave the care system and move into independent living, in order to take back control over their lives.

I have no control over my own life. [16yo]

I want to go into independent living, because I've had enough of living with, like, parent figures and I just want to be on my own and be independent and prove that I can do it. [16yo]

Theme 2: Social support was considered key to coping but not always available, while access to professional support was inconsistent. The majority of participants most valued

For peer review only - http://bmjopen.bmj.com/site/about/guidelines.xhtml

informal day-to-day support from a trusted adult, while describing formal/professional support to be less available and less helpful. Many participants expressed the need for professional support to be improved and made more readily available.

Subtheme 1: Day-to-day support from a trusted adult was important for wellbeing. Having a trusted adult to talk to about day-to-day problems or concerns, whenever they felt they needed to, was identified as the most valuable form of support for participants. Particular aspects valued in a trusted adult were their ability to listen, to *"keep secrets"* and to *"give good advice".* The importance of being able to call upon this support as and when it was needed was often emphasised. For example, participants described the benefits of being able to talk to carers after a bad day or to speak with key staff at school if there was an argument in a lesson. Knowing there was always a trusted adult available to speak to was central to maintaining wellbeing for many participants.

> *Telling* [your worries/concerns to] *someone you trust makes you a lot calmer*. [16yo]

Overall, carers were by far reported as the primary source of support from the perspective of most of the participants. Some participants also talked about their social worker or a school staff member being key sources of support.

> *..most foster carers are very nice and they really help you with your life… they look after and like you.* [10yo]

> *Whenever something* [is] *tough, I'll tell her* [social worker] *and she'd always take me out and then we'd always chat about something different and then she'd take my mind off it.* [16yo]

> *My school nurse helped me a lot because whenever I had a problem I'd go to her and she'd try to sort it out as quick as possible.* [16yo]

While there were some cases of very positive relationships between a young person and their social worker, perceived ineffective communication between social workers and young

For peer review only - http://bmjopen.bmj.com/site/about/guidelines.xhtml

people was identified as one of their central problems in terms of feeling supported. A focus of many interviews was how transient their social worker was, with almost all having multiple different social workers over their time in care, or even over the past year. This transience made it particularly difficult for young people to build trust, which could also influence their willingness to disclose information about their early experiences and their general trust of professionals. Some also described situations in which they viewed that confidentiality had been breached by adults in their support network, further resulting in them feeling let down and trust being corroded.

You don't know who's coming and going. [16yo]

I just want one […] one person [Social Worker] who is going to be there for quite a long time and not going to leave all of a sudden and I can build up a bond. [15yo]

You tell them [about your experiences] and then they leave and you can't tell them anymore. So you're like ohmygod who can I tell? Who can I trust? It's hard. [16yo]

A minority of participants perceived that there was no one they could talk to. These participants were all adolescents and all reported struggling with significant mental health difficulties, including engaging in self-harm and/or drug or alcohol use. For these young people a lack of a trusted adult, which largely included an unstable placement, was particularly problematic. Perceived unhelpful foster placements were discussed in terms of young people perceiving carers fostering *"for the money",* blaming or not trusting the young person, not putting effort in to spending time with them, or the young person perceiving that the carer had not been appropriately trained.

Since I was seven all I've ever done is either sat on the sofa and watched tv on my own coz no one talks to me or I've sat in my room and watched tv on my own because nobody ever talks to me [16yo]

For peer review only - http://bmjopen.bmj.com/site/about/guidelines.xhtml

Subtheme 2: Formal mental health support was often unavailable and opinions on usefulness were mixed. Formal support from mental health professionals was identified as being often unavailable and unhelpful. For those who had seen a mental health professional, this was often the school counsellor, with the minority accessing formal child and adolescent mental health services (CAMHS). Among those who had received support, either in the form of a school counsellor or CAMHS, opinions were mixed with a slight majority describing it as unhelpful. Young people had difficultly specifically identifying what they found unhelpful about this support, although some did describe feeling restricted to a scheduled appointment and having to talk about their past despite not wanting to do so.

> *It made it worse like, having a scheduled time to go and talk about things when I don't really want to talk about them, like I'd rather just say it when I felt like saying it. It just made me think about it more which made it worse.* [14yo]

Similarly, those who had positive experiences with mental health professionals struggled to identify what it was that had been helpful, but just that it provided them a safe space to talk and to help them cope.

> *It made me talk to people and like actually not just keep everything to yourself.* [15yo]
>
> *I got my feelings clear to everyone.* [11yo]

Theme 3: Talking about problems was seen as important, but maladaptive coping strategies were also reported.

When participants were asked what they do when experiencing negative emotions, a range of coping strategies were identified. Some of these strategies were adaptive, such as talking to somebody they trusted, and some were maladaptive, such as self-harm.

Subtheme 1: Talking about worries or challenges was identified as a helpful strategy for coping with negative emotions. Talking about day-to-day worries or problems with a trusted person was identified as a common strategy used in response to negative

For peer review only - http://bmjopen.bmj.com/site/about/guidelines.xhtml

emotions, such as sadness, anger, or anxiety. As previously highlighted, carers, teachers and social workers were amongst those who participants felt comfortable speaking to. This was discussed as a primarily helpful strategy, often resulting in feeling better afterwards or enabling problems to be resolved.

If you're feeling down, always talk to someone that you're close to about it, never hold it in. [16yo]

Subtheme 2: A majority avoided talking about their pre-care experiences and in the context of mental health difficulties maladaptive coping was common. While talking to someone about current worries was seen as important for coping, most participants thought it was best to avoid talking about their pre-care experiences. Indeed, when asked what advice they might give a friend who was struggling, many endorsed they would encourage them to talk to someone, but then also endorsed that this was not something they themselves did.

Speak up and tell people how you are feeling and what you've been through, and that you're struggling to cope. Because if people don't know people can't help.

Interviewer: Did you do that?

No, because I didn't know them and I don't trust people I don't know. [16yo]

Reasons for avoidance of discussing their maltreatment varied but the most common justifications were either because talking about pre-care experiences made them feel uncomfortable or because they did not think it would be helpful. This sense of discomfort when talking about previous experiences often appeared to be associated with a lack of trust in adults and services. Participants also discussed feeling embarrassed, uncomfortable with the amount of attention placed on them, and unsure of how to act when describing events.

I don't really like talking about my past too much, it's, it's like all attentions on me and it's like awkward. [14yo]

For peer review only - http://bmjopen.bmj.com/site/about/guidelines.xhtml

It don't change nothing, it don't mean it go back. It still happened you can't make it [go away]. [10yo]

From those who identified talking as an unhelpful strategy, many held the attitude that, as events were in the past, talking about them would not change anything. Some also felt that such discussion acted as a barrier to moving forward. This was particularly reported by young people who were experiencing serious mental health difficulties, including current distressing intrusive memories of their maltreatment.

I'm not saying "suck it up and be a man" but just try and just keep your mind off it, keep doing what you're doing cos you don't really want to let the past like take over you. Because the past is in the past and I really don't want to bring my past up. I'm trying to move forward for the minute but obviously if people are bringing it up, I can't. [15yo]

In general, many participants endorsed avoidance of maltreatment memories and avoidance of talking about their past as a primary way to cope. In most cases this involved distracting themselves with activities, such as watching TV and spending time with friends. Indeed in many cases when talking about their support systems, young people endorsed that adults helping to distract them was helpful (e.g changing the topic; social workers taking them to lunch; see Theme 2 quotes). These distractions shifted the focus of their attention onto something else, allowing them to avoid thinking about their early experiences.

[in response to question about coping] *just watch tv or something.* [10yo]

I would go on the PlayStation and just kill people. [15yo]

In the case of adolescents who were struggling with mental health difficulties, there were more frequent examples of destructive coping strategies, particularly self-harm and drug and alcohol use. Many of these young people reported that they did not have any other way of coping with their emotional difficulties.

For peer review only - http://bmjopen.bmj.com/site/about/guidelines.xhtml

I pretend to be someone I'm not. I pretend to be okay when I'm not and let emotions build up until eventually I just break. [12yo]

Getting drunk a lot, doing a lot of drugs. [14yo]

I cry and I hurt myself that's literally the only way I know how to deal with things anymore. [16yo]

Discussion

This study aimed to learn more about the views of young people in out-of-home care, in relation to coping, support, and help-seeking with emotional difficulties. Across the interviews there were core common themes, including the importance of having a consistent and trusted adult with whom they could share worries. Carers were identified as the primary source of emotional support by most young people. While many participants talked about the importance of talking to your support system as a means of coping, most also thought that it was not helpful to talk about their pre-care experiences. Adolescents experiencing mental health difficulties also commonly struggled to identify adaptive coping strategies or support systems.

Similar to other qualitative work with young people in care [9,12], participants viewed the support of a trusted adult as key to coping with daily stress and interpersonal difficulties, and with more pervasive mental health difficulties. In the vast majority of cases, the carer (or a key worker if in residential care) was viewed as the primary source of this support, although some young people talked about this support also coming from their social worker, a trusted staff member at school, or friend. Trustworthiness was discussed as particularly central to the young person feeling supported, including the perception that the adult would be able to keep information confidential. A lack of trust was often expressed as a key concern and potential barrier to seeking help. While some young people had positive relationships with their social workers, many participants discussed changes in social workers

For peer review only - http://bmjopen.bmj.com/site/about/guidelines.xhtml

as another key barrier to building a trusted relationship and seeking support for emotional difficulties. This reflects similar findings by Wood and Selwyn in their qualitative work, where young people in care also discussed the challenges of navigating regular changes in social workers and the ultimate perceived negative impact on their subjective wellbeing [25]. Findings highlight the need for services to explore policies around mitigating some of the consequences of social worker changes on the young person's wellbeing. Social work has some of the highest burn-out rates of any profession. Providing more robust training and support to social workers is considered key to reducing social work changes [26]. However, given some change is inevitable, policies for how these changes happen are crucial, to minimise the potential eroding of trust.

While many participants talked about going to their carer for support if they were feeling worried or upset (e.g., if they were upset about something at school), many at the same time emphatically stated that they did not think it was useful to talk about their maltreatment experiences, with either their carer or mental health professionals. While this was the case across much of the sample, it is perhaps most challenging for those young people who discussed experiencing significant mental health difficulties. This sub-set of the sample most commonly reported feeling that they had no person they could turn to for support, that they did not want to talk to a professional about their maltreatment or mental health, and had no strategies for coping with their mental health beyond self-harm and drug and alcohol abuse. Beliefs around not talking about maltreatment or trauma present a particular challenge to mental health and social-care professionals. Of course, young people could and should not be forced to talk about traumatic experiences. Some may also be coping fine and not need to talk through their experiences. It is also completely understandable that young people may not want to talk about distressing early experiences. Yet, avoiding talking or thinking about traumatic experiences (i.e., thought suppression) is a key maintainer of poor

For peer review only - http://bmjopen.bmj.com/site/about/guidelines.xhtml

mental health outcomes [15], including for young people in care [16]. Further, a key component of evidenced-based trauma-focused psychological support involves processing the traumatic experiences, commonly via a trauma narrative [e.g., 27]. There remains an urgent need to understand how services can best engage young people in care who may benefit from professional support in treatments that might involve talking about their experiences, particularly for a group where developing trust and a strong therapeutic relationship is essential [10]. This may be partly achieved by having systems, including at a social-care and education level, where staff are appropriately trained and supported in engaging with these challenging conversations with young people from earlier on in their time in care, so young people know that it is safe to talk about these experiences if they want to. Many services in the UK would report using "trauma-informed" approaches to their practice [28], which would include creating safe spaces for young people to talk about their experiences. However, the evidence-base for how these approaches are genuinely implemented and their effectiveness, remains scarce. Relatedly, many young people reported feeling stigmatised by being in care, including by professionals (e.g., teachers) and peers – a finding that is also in line with other qualitative work with this group [e.g., 25]. It will therefore also be important for services to consider how to address issues of stigma, which can be a barrier to help-seeking. Strength and relationship-based approaches may be useful here [29], but remain poorly researched in terms of how they are used in practice or their general effectiveness. These remain important areas for future research, to focus not just on targeting psychopathology but more broadly improving the emotional wellbeing of these young people.

For the minority of participants in this study who had received professional mental health support, including via CAMHS and school counselling, views on this support were mixed. In their 2008 review of qualitative literature on children in care and mental health services, Davies and Wright highlighted that many young people felt wary and unsure of

For peer review only - http://bmjopen.bmj.com/site/about/guidelines.xhtml

professional mental health support, and they highlighted the importance of developing trust [9]. Ten years later, the current study found that young people in care are still experiencing these same concerns. There remains an urgent need to improve understanding of the service-level barriers to accessing professional support, including access to evidence-based psychological interventions (e.g., see review [30]). Within the current UK CAMHS system, thresholds for professional mental health support often mean young people are in crisis, with complex mental health needs, by the time they can access support (e.g., see [31]). Exploring feasible but appropriate avenues for providing effective evidence-based support, before reaching crisis, is clearly an important avenue for addressing the mental health needs of these young people.

Limitations

While this study has key strengths, including a focus on an under-researched group, and the use of qualitative interviews to provide an in-depth exploration of their views on coping and wellbeing, findings should also be considered in light of key limitations. First, we used opportunity sampling methods. All young people were participants in a larger quantitative study of mental health and wellbeing. Young people who did not want to talk about their experiences and mental health would likely have declined participating in this study. Participants also all came from two local authorites in England. While findings cannot necessarily be generalised to other young people in out-of-home care, it is worth noting the significant overlap between these findings and qualitative work with young people in care and foster carers in the UK, US, and Australia [e.g., 9,19]. Finally, quantitative research is needed to provide more robust evidence on the consequences of different support systems and coping strategies in relation to the mental health of these young people.

Conclusions

For peer review only - http://bmjopen.bmj.com/site/about/guidelines.xhtml

For peer review only

In sum, while there were positives to being in care and many young people felt well supported by their carer, results of this qualitative study identified key areas to be addressed to improve support for the emotional wellbeing of this group. This included improving the continuity of social workers and carers to allow young people to develop trusted relationships with key adults, targeting broader concerns around stigma and trust (including in the school system) that may act as a barrier to help-seeking, and ensuring young people are receiving appropriate evidence-based psychological support in a more timely manner, before reaching crisis point. Addressing these issues remains a significant challenge for services, particularly alongside long-term underfunding of mental health services. Yet, failing to provide adequate support, both in terms of informal day-to-day support and professional services, can have a substantial impact on the trajectory of these young people.

For peer review only

Contributors: RMH is the lead researcher on the project and PI on the ESRC grant. She led the conceptualisation of the project, conducted a proportion of the interviews, and was responsible for overall project management. SLH was involved in the conceptualisation of the project, including the design of the interview schedule and also contributed to discussion on the themes and feedback on the manuscript. RM-S was involved in the conceptualisation of the project and contributed to the discussion about the themes and feedback on the manuscript. EE was the research assistant on the project and conducted a proportion of the interviews and contributed to transcribing, data analysis, and feedback on the manuscript. ER-E was involved in the transcribing, contributed to data analysis and provided feedback on the manuscript. TH contributed to transcribing and feedback on the codes and themes.

The authors also wish to sincerely thank the many social-care and mental health service staff, carers, and young people who contributed to the design of this project and from whom we continue to learn so much.

Funding: This research was funded by an ESRC Future Leader Grant (ES/N01782X/1) and University of Bath Prize Fellowship awarded to RMH.

Competing Interests: None to declare.

Data sharing: Anonymised transcripts will be available on request on a case-by-case basis.

For peer review only

References

[1] Department for Education. Statistics: looked after children. London: Author. 2018.

[2] Pecora PJ, White CR, Jackson LJ, Wiggins T. Mental health of current and former recipients of foster care: A review of recent studies in the USA. Child & Family Social Work. 2009; 14(2):132-146.

[3] Hiller RM, St Clair MCS. The emotional and behavioural symptom trajectories of children in long-term out-of-home care in an English local authority. Child Abuse & Neglect. 2018; 81:106-117.

[4] Newton RR, Litrownik AJ, Landsverk JA. Children and youth in foster care: Disentangling the relationship between problem behaviors and number of placements. Child Abuse & Neglect. 2000; 24(10):1363-1374.

[5] Ford T, Vostanis P, Meltzer H, Goodman R. Psychiatric disorder among British children looked after by local authorities: comparison with children living in private households. The British Journal of Psychiatry. 2007; 190(4):319-325.

[6] McMillen JC, Zima BT, Scott Jr LD, Auslander WF, Munson MR, Tollie MT, Spitznagel EL. Prevalence of psychiatric disorders among older youths in the foster care system. Journal of the American Academy of Child & Adolescent Psychiatry. 2005; 44(1):88-95.

[7] Oswald SH, Heil K, Goldbeck L. History of maltreatment and mental health problems in foster children: A review of the literature. Journal of Pediatric Psychology. 2009; 35(5):462-472.

[8] Jones R, Everson-Hock ES, Papaioannou D, Guillaume L, Goyder E, Chilcott J, ... & Swann C. Factors associated with outcomes for looked-after children and young people: a correlates review of the literature. Child: Care, Health and Development. 2011; 37(5):613-622.

For peer review only - http://bmjopen.bmj.com/site/about/guidelines.xhtml

[9] Davies J, Wright J. Children's voices: A review of the literature pertinent to looked-after children's views of mental health services. Child and Adolescent Mental Health. 2008; 13(1):26-31.

[10] Shaw M, De Jong M. Child abuse and neglect: a major public health issue and the role of child and adolescent mental health services. The Psychiatrist. 2012; 36(9):321-325.

[11] Ahmed K, Windsor L, Scott S. In their own words: abused children's perceptions of care provided by their birth parents and foster carers. Adoption & Fostering. 2015; 39(1): 21-37.

[12] Sinclair I, Wilson K. Matches and mismatches: The contribution of carers and children to the success of foster placements. British Journal of Social Work. 2003; 33(7):871-884.

[13] Rock S, Michelson D, Thomson S, Day C. Understanding foster placement instability for looked after children: A systematic review and narrative synthesis of quantitative and qualitative evidence. British Journal of Social Work. 2013; 45(1):177-203.

[14] Murray L, Creswell C, Cooper PJ. The development of anxiety disorders in childhood: an integrative review. Psychological Medicine. 2009; 39(9):1413-1423.

[15] Trickey D, Siddaway AP, Meiser-Stedman R, Serpell L, Field AP. A meta-analysis of risk factors for post-traumatic stress disorder in children and adolescents. Clinical Psychology Review. 2012; 32(2):122-138.

[16] Hiller RM, Meiser-Stedman R, Elliott E, Banting R, Halligan SL. Cognitive predictors of (complex) posttraumatic stress: A longitudinal study of young people in out-of-home care. Journal of Child Psychology and Psychiatry. 2020; In Press.

[17] Marshall B, Cardon P, Poddar A, Fontenot R. Does sample size matter in qualitative research?: A review of qualitative interviews in IS research. Journal of computer information systems. 2013; 54(1):11-22.

[18] Vasileiou K, Barnett J, Thorpe S, Young T. Characterising and justifying

For peer review only - http://bmjopen.bmj.com/site/about/guidelines.xhtml

sample size sufficiency in interview-based studies: systematic analysis of qualitative health research over a 15-year period. BMC Medical Research Methodology. 2018; 18(1):148.

[19] Ahrens KR, DuBois DL, Garrison M, Spencer R, Richardson LP, Lozano P. Qualitative exploration of relationships with important non-parental adults in the lives of youth in foster care. Children and Youth Services Review. 2011; 33(6):1012-1023.

[20] Johnson E, Menna R. Help seeking among adolescents in foster care: A qualitative study. Children and Youth Services Review. 2017; 76:92-99.

[21] Docherty S, Sandelowski M. Focus on qualitative methods: Interviewing children. Research in Nursing & Health. 1999; 22(2):177-185.

[22] Van Wesel F, Boeije H, Alisic E, Drost S. I'll be working my way back: A qualitative synthesis on the trauma experience of children. Psychological Trauma: Theory, Research, Practice, and Policy. 2012; 4(5):516.

[23] Braun V, Clarke V. Using thematic analysis in psychology. Qualitative Research in Psychology. 2006; 3(2):77-101.

[24] Braun V, Clarke V, Hayfield N, Terry G. Thematic analysis. In P Liamputtong. Handbook of Research Methods in Health Social Sciences. Singapore: Springer. 2019. 843-60.

[25] Wood M, Selwyn J. Looked after children and young people's views on what matters to their subjective well-being. Adoption & Fostering. 2017; 41(1):20-34.

[26] Kim H, Ji J, Kao D. Burnout and physical health among social workers: A three-year longitudinal study. Social Work. 2011; 56(3):258-268.

[27] Deblinger E, Mannarino AP, Cohen JA, Runyon MK, Steer RA. Trauma-focused cognitive behavioral therapy for children: impact of the trauma narrative and treatment length. Depression and Anxiety. 2011; 28(1):67-75.

For peer review only - http://bmjopen.bmj.com/site/about/guidelines.xhtml

[28] Beyerlein BA, Bloch E. Need for trauma-informed care within the foster care system: A policy issue. Child Welfare. 2014; 93(3):7.

[29] Brooks R, Brooks S. Creating resilient mindsets in children and adolescents: A strength-based approach for clinical and nonclinical populations. In Resilience interventions for youth in diverse populations. Springer, New York, NY. 2014; 59-82.

[30] Leve LD, Harold GT, Chamberlain P, Landsverk JA, Fisher PA, Vostanis P. Practitioner review: children in foster care–vulnerabilities and evidence-based interventions that promote resilience processes. Journal of Child Psychology and Psychiatry. 2012; 53(12): 1197-1211.

[31] Blow E. Children and young people's mental health – policy, CAMHS services, funding and education. London: House of Commons Library. 2015.

For peer review only

Table 1. *Sample demographics.*

**Young person descriptives (N = 25)**	
Age, *M* (*SD*), Range	13.0 (2.24)
Sex (% female)	56%
Ethnicity (% Caucasian)	80%
Age entered care, *M* (*SD*)	8.7 yrs (4.2)
Time in care, *M* (*SD*)	4.2 yrs (3.6)
% who had multiple placements	80%
Type of placement	
Non-biological foster carer	76%
Biological foster carer (e.g., grandmother)	16%
Residential care home	8%

For peer review only

For peer review only - http://bmjopen.bmj.com/site/about/guidelines.xhtml

Table 2. *Themes and subthemes from thematic analysis.*

Themes	Sub-themes
**Being in care could create a sense of safety but some young people struggled with a lack of control and agency**	Being in care can create a sense of safety and of being part of a family.
	Negative aspects of being in care included feeling different and not being listened to or respected.
**Social support was considered key to coping but not always available, while access to professional support was inconsistent.**	Day-to-day support from a trusted adult was identified as very important for wellbeing.
	Formal mental health support was often unavailable and opinions on usefulness were mixed.
**Talking about problems was seen as important, but maladaptive coping strategies were also reported**	Talking about worries or challenges was identified as a helpful strategy for coping with negative emotions.
	A majority avoided talking about their pre-care experiences and in the context of mental health difficulties maladaptive coping was common.

For peer review only

For peer review only - http://bmjopen.bmj.com/site/about/guidelines.xhtml

Child qualitative interview

Thank you for talking to me today. Like it said in the letter I'm interested in finding out a bit more about your views on how you cope and where you might go for support or help if you're struggling with thoughts or feelings. Remember, you don't need to talk about your experiences in your home. You can if you want to but we won't be asking you to. What I'm really interested in is what happened *after* this, when you came in to care. This isn't like a test at school, there are no right or wrong answers, it's just about talking honestly and sharing your opinion. If you want to stop at any time or take a break just let me know. Does that sound ok?

Demographics

- Do you remember how old you were when you first came in to care?
- How long have you lived in [current placement]?

Reactions

- How have you felt since you came in to care?
  - Are there good parts?
  - What are some of the harder parts?

Support

- If you're having a hard time, is there anything you do to try and make yourself feel better or different?
  - (*example might be argument with friend at school; through to mental health difficulty if yp has disclosed this*)

- Is there anyone you talk to, to try to make yourself feel better?
  - Who? Is that helpful/unhelpful? Why?
  - Anyone else?
  - Have you ever talked to a professional? Like a psychologist or school counsellor? What was this like? What was helpful/unhelpful?

- Do you ever talk to anyone about what you experienced before you came into care?
  - Why/why not?
  - What helps/doesn't help?

Support wishes

- What support do you wish you had got that you didn't?
  - (example might be – *if you were asked to create the best possible care system to support young people who were struggling with their thoughts and feelings, what would it look like?*)
- If you had a friend that went through the same things as you, how would you help them? What advice might you give them to help them cope with their thoughts and feelings?

Is there anything else you want to add that we haven't covered?

For peer review only - http://bmjopen.bmj.com/site/about/guidelines.xhtml

Reporting checklist for qualitative study.

Based on the SRQR guidelines.

Instructions to authors

Complete this checklist by entering the page numbers from your manuscript where readers will find each of the items listed below.

Your article may not currently address all the items on the checklist. Please modify your text to include the missing information. If you are certain that an item does not apply, please write "n/a" and provide a short explanation.

Upload your completed checklist as an extra file when you submit to a journal.

In your methods section, say that you used the SRQRreporting guidelines, and cite them as:

O'Brien BC, Harris IB, Beckman TJ, Reed DA, Cook DA. Standards for reporting qualitative research: a synthesis of recommendations. Acad Med. 2014;89(9):1245-1251.

		Reporting Item	Page Number
**Title**			
	#1	Concise description of the nature and topic of the study identifying the study as qualitative or indicating the approach (e.g. ethnography, grounded theory) or data collection methods (e.g. interview, focus group) is recommended	1

For peer review only

Abstract

	#2	Summary of the key elements of the study using the abstract format of the intended publication; typically includes background, purpose, methods, results and conclusions	2

Introduction

Problem formulation	#3	Description and signifcance of the problem / phenomenon studied: review of relevant theory and empirical work; problem statement	4-5
Purpose or research question	#4	Purpose of the study and specific objectives or questions	5

Methods

Qualitative approach and research paradigm	#5	Qualitative approach (e.g. ethnography, grounded theory, case study, phenomenolgy, narrative research) and guiding theory if appropriate; identifying the research paradigm (e.g. postpositivist, constructivist / interpretivist) is also recommended; rationale. The rationale should briefly discuss the justification for choosing that theory, approach, method or technique rather than other options available; the assumptions and limitations implicit in those choices and how those choices influence study conclusions and transferability.	7-8

For peer review only

		As appropriate the rationale for several items might be discussed together.	
Researcher characteristics and reflexivity	#6	Researchers' characteristics that may influence the research, including personal attributes, qualifications / experience, relationship with participants, assumptions and / or presuppositions; potential or actual interaction between researchers' characteristics and the research questions, approach, methods, results and / or transferability	7
Context	#7	Setting / site and salient contextual factors; rationale	6
Sampling strategy	#8	How and why research participants, documents, or events were selected; criteria for deciding when no further sampling was necessary (e.g. sampling saturation); rationale	6
Ethical issues pertaining to human subjects	#9	Documentation of approval by an appropriate ethics review board and participant consent, or explanation for lack thereof; other confidentiality and data security issues	6
Data collection methods	#10	Types of data collected; details of data collection procedures including (as appropriate) start and stop dates of data collection and analysis, iterative process, triangulation of sources / methods, and modification of procedures in response to evolving study findings; rationale	6

For peer review only

Data collection instruments and technologies	#11	Description of instruments (e.g. interview guides, questionnaires) and devices (e.g. audio recorders) used for data collection; if / how the instruments(s) changed over the course of the study	6-8
Units of study	#12	Number and relevant characteristics of participants, documents, or events included in the study; level of participation (could be reported in results)	6-8
Data processing	#13	Methods for processing data prior to and during analysis, including transcription, data entry, data management and security, verification of data integrity, data coding, and anonymisation / deidentification of excerpts	6-8
Data analysis	#14	Process by which inferences, themes, etc. were identified and developed, including the researchers involved in data analysis; usually references a specific paradigm or approach; rationale	6-8
Techniques to enhance trustworthiness	#15	Techniques to enhance trustworthiness and credibility of data analysis (e.g. member checking, audit trail, triangulation); rationale	7-8
**Results/findings**			
Syntheses and interpretation	#16	Main findings (e.g. interpretations, inferences, and themes); might include development of a theory or model, or integration with prior research or theory	8-16

For peer review only

Links to empirical data	#17	Evidence (e.g. quotes, field notes, text excerpts, photographs) to substantiate analytic findings	8-16
**Discussion**			
Intergration with prior work, implications, transferability and contribution(s) to the field	#18	Short summary of main findings; explanation of how findings and conclusions connect to, support, elaborate on, or challenge conclusions of earlier scholarship; discussion of scope of application / generalizability; identification of unique contributions(s) to scholarship in a discipline or field	16
Limitations	#19	Trustworthiness and limitations of findings	19
**Other**			
Conflicts of interest	#20	Potential sources of influence of perceived influence on study conduct and conclusions; how these were managed	21
Funding	#21	Sources of funding and other support; role of funders in data collection, interpretation and reporting	21

Notes:

- 21: n/a (blinded) The SRQR checklist is distributed with permission of Wolters Kluwer © 2014 by the Association of American Medical Colleges. This checklist was completed on 30. July 2019 using https://www.goodreports.org/, a tool made by the EQUATOR Network in collaboration with Penelope.ai

For peer review only

Coping and support-seeking in out-of-home care: A qualitative study of the views of young people in care in England

Journal:	*BMJ Open*
Manuscript ID	bmjopen-2020-038461.R3
Article Type:	Original research
Date Submitted by the Author:	13-Dec-2020
Complete List of Authors:	Hiller, Rachel; University of Bath, Department of Psychology<br>Halligan, Sarah; University of Bath, Psychology; University of Cape Town, Department of Psychiatry and Mental Health<br>Meiser-Stedman , Richard ; University of East Anglia,<br>Elliott, Elizabeth; University of Bath, Department of Psychology; University of Reading, School of Psychology and Clinical Language Science<br>Rutter-Eley, Emily; University of Reading, School of Psychology and Clinical Language Science<br>Hutt, Tilly; University of Bath, Department of Psychology
**Primary Subject Heading**:	Mental health
Secondary Subject Heading:	Qualitative research, Paediatrics
Keywords:	Child protection < PAEDIATRICS, Child & adolescent psychiatry < PSYCHIATRY, Quality in health care < HEALTH SERVICES ADMINISTRATION & MANAGEMENT

I, the Submitting Author has the right to grant and does grant on behalf of all authors of the Work (as defined in the below author licence), an exclusive licence and/or a non-exclusive licence for contributions from authors who are: i) UK Crown employees; ii) where BMJ has agreed a CC-BY licence shall apply, and/or iii) in accordance with the terms applicable for US Federal Government officers or employees acting as part of their official duties; on a worldwide, perpetual, irrevocable, royalty-free basis to BMJ Publishing Group Ltd ("BMJ") its licensees and where the relevant Journal is co-owned by BMJ to the co-owners of the Journal, to publish the Work in this journal and any other BMJ products and to exploit all rights, as set out in our licence.

The Submitting Author accepts and understands that any supply made under these terms is made by BMJ to the Submitting Author unless you are acting as an employee on behalf of your employer or a postgraduate student of an affiliated institution which is paying any applicable article publishing charge ("APC") for Open Access articles. Where the Submitting Author wishes to make the Work available on an Open Access basis (and intends to pay the relevant APC), the terms of reuse of such Open Access shall be governed by a Creative Commons licence – details of these licences and which Creative Commons licence will apply to this Work are set out in our licence referred to above.

Other than as permitted in any relevant BMJ Author's Self Archiving Policies, I confirm this Work has not been accepted for publication elsewhere, is not being considered for publication elsewhere and does not duplicate material already published. I confirm all authors consent to publication of this Work and authorise the granting of this licence.

For peer review only

Coping and support-seeking in out-of-home care: A qualitative study of the views of young people in care in England

Rachel M Hiller[1], Sarah L Halligan[1,2], Richard Meiser-Stedman[3], Elizabeth Elliott[1,4], Emily Rutter-Eley[4], Tilly Hutt[1]

[1] Department of Psychology, University of Bath, UK

[2] Department of Psychiatry and Mental Health, University of Cape Town, South Africa

[3] Department of Clinical Psychology, Norwich Medical School, University of East Anglia, UK

[4] School of Psychology and Clinical Language Science, University of Reading, UK

* Corresponding Author:

Dr Rachel Hiller

Department of Psychology

University of Bath

P. 01225 383794

E. R.Hiller@bath.ac.uk

Main text word count: 5, 755

For peer review only

For peer review only - http://bmjopen.bmj.com/site/about/guidelines.xhtml

Abstract

Objectives: Young people who have been removed from their family home and placed in out-of-home care have commonly experienced abuse, neglect and/or other forms of early adversity. High rates of mental health difficulties have been well-documented in this group. The aim of this research was to explore the experiences of these young people within the care system, particularly in relation to support-seeking and coping with emotional needs, to better understand feasible and acceptable ways to improve outcomes for this group.

Design and Study-Setting: This study used 1:1 semi-structured qualitative interviews with young people in out-of-home care in England, to provide an in-depth understanding of their views of coping and support for their emotional needs, both in terms of support networks and experiences with mental health services. Participants were 25 10-16 years olds (56% female) and included young people living with non-biological foster carers, kinship carers, and in residential group homes.

Results: Participants described positive (e.g. feeling safe) and negative (e.g. feeling judged) aspects to being in care. Carers were identified as the primary source of support, with a supportive adult central to coping. Views on support and coping differed for young people who were experiencing more significant mental health difficulties, with this group largely reporting feeling unsupported and many engaging in self-harm. The minority of participants had accessed formal mental health support, and opinions on usefulness were mixed.

Conclusions: Results provide insight, from the perspective of care-experienced young people, about both barriers and facilitators to help-seeking, as well as avenues for improving support.

Key words: child maltreatment; care-experienced; adolescents; foster care; emotional wellbeing; mental health; help-seeking; qualitative

For peer review only

Strengths and Limitations of the Study:

- This study is focused on young people living in out-of-home care, representing an under-researched group where there remains significant need.
- Using 1:1 qualitative interview methods we gained an in-depth understanding of the views and experiences of these young people, in terms of coping and help-seeking around mental health.
- We used opportunity sampling methods, which may introduce sampling bias
- As this study uses qualitative methods, we cannot conclude whether different types of reported coping might be associated with better or worse outcomes.

For peer review only

Introduction

The most common reason a child would be removed from their home and placed in out-of-home care is due to the experience of abuse and/or neglect [e.g., 1,2]. In the UK, it is also the case that most young people who enter care are removed from their home when school-aged or older, meaning early experiences of maltreatment have often been ongoing for a number of years [1]. Once in care many continue to face ongoing instability. Separation from siblings is common, as are moves between different placement providers, resulting in the potential for inconsistent support [3-4]. Given these accumulative experiences, it is perhaps unsurprising that there is well-established evidence that young people in out-of-home care experience high rates of psychological difficulties compared to their peers [5-7].

Failing to adequately address the psychological or emotional needs of young people in care has been identified as a key driver of many of the well-documented poor outcomes that can categorise this group, including school attainment and later rates of homelessness and unemployment [8]. Yet, despite the long-documented poor outcomes, there has been limited evidence of improvements in the overall picture of mental health for children in care in the UK over the past decade [1]. One concern is that these young people can fall between the gaps of children's social-care and national health service provisions, which can result in poor access to mental health services [9-10]. With resource issues affecting child and adolescent mental health services (CAMHS) in many countries, it is also largely impossible for these services to address the large number of young people in care who may benefit from professional mental health support.

In the face of potentially limited professional support services, young people will inevitably develop day-to-day coping strategies and support networks – whether perceived to be adaptive or maladaptive. Some qualitative work has investigated the views of young people in care in relation to support networks, and specifically their relationships with carers,

For peer review only - http://bmjopen.bmj.com/site/about/guidelines.xhtml

finding developing trust, consistent boundaries, and empathy were viewed as important [11-12]. However, there is less evidence for how they may or may not engage with other support networks, including peers, school, and social workers. Such evidence is potentially particularly important for this group, given one-third of young people in care in the UK will have more than one carer in any given year [1], and given the well-documented cyclical association between placement breakdowns and emotional difficulties [4,13]. Beyond the role of external support networks, we know little about what strategies young people in care may be using themselves to manage their emotional wellbeing. While many young people in care struggle with emotional difficulties, it is important to note that not all do. Evidence from the wider child mental health field shows that engaging in certain types of coping strategies can be associated with better or worse mental health difficulties [14-15], including in care-experienced young people [16]. Understanding how young people in care cope with emotional difficulties, particularly in the face of potentially disrupted support networks, is crucial to understanding both the experiences of this group, and the avenues for improving formal and informal support.

Using a semi-structured qualitative approach we sought to explore the following questions: (1) where do young people in care seek support for emotional difficulties, both in terms of social support and professional services?; (2) what do they view as barriers to seeking help?; and (3) what coping strategies do they use when experiencing emotional difficulties? This study included a focus on more day-to-day coping and help-seeking (e.g., if they felt stressed) and coping and help-seeking around more significant mental health difficulties.

Method

Recruitment and Sample

For peer review only - http://bmjopen.bmj.com/site/about/guidelines.xhtml

Ethical approval was granted from the University of Bath Psychology- and Social Care- research ethics committees, along with permission from the participating local authorities. We recruited 25 young people aged 10 – 16 years old. Participants were all part of a larger quantitative study on the mental health and wellbeing of young people in care [16]. We used opportunity sampling, with consecutive young people invited to participate in an optional qualitative interview alongside their participation in the larger study. The full procedures for the larger study can be found in reference [16]. The sample size was based on general guidance for qualitative methodology [see 17-18]. While clear guidance for sample sizes in qualitative research remains limited, our sample size was chosen to be in line with, or larger than, similar studies in the field [e.g., 19-20]. It was also based on our previous qualitative work with young people in terms of the sample required to reach saturation, with capacity to continue recruitment if it was felt saturation was not reached or the data was inadequate (which was ultimately not the case). The sample size also allowed us to explore views of older and younger children within our age range, and include young people with a range of mental health needs, from those who were managing well to those struggling with chronic mental health difficulties.

Participants came from two local authorities in England. Based on national data, one was a smaller-sized local authority and one was medium-sized, with both in largely urban areas. Informed consent for the young person's participation was provided by their social worker, while the young person provided informed assent (or consent if 16 years old). Eligibility for participation in the quantitative study were: aged 10-17 years old, on full care order if under 16 years old (where the local authority has acquired parental responsibility for the young person), absence of developmental disability that would preclude the child from mainstream schooling, and absence of significant current risk (e.g., current severe suicidality). Young people could be in any style of placement, including foster placement

For peer review only - http://bmjopen.bmj.com/site/about/guidelines.xhtml

with a non-biological carer, kinship placement, residential care home. Descriptive information on the sample are presented in Table 1.

Procedure and Measures

In-person 1:1 interviews were conducted by two researchers. The lead researcher (RH) had prior training and experience in qualitative research, while the other (EE) was experienced researching with vulnerable young people and adults and received further training specifically on qualitative procedures. We used a 1:1 semi-structured interview format, rather than focus group, due to the potential for sensitive information being discussed that young people might not have wished to disclose in a group. For one of these interviews two siblings requested to complete the interview together, while for two interviews the carer remained present at the request of the young people. All others (n = 22) had only the young person and interviewer present. RH and EE met regularly throughout the study to monitor the quality of the interviews and reflect on the content. All interviews were audio recorded and later transcribed verbatim. As a thank you for volunteering their time, the young people each received a £10 voucher. As per standard risk management and safeguarding procedures, and where appropriate, summary letters were provided to social workers to support potential referrals to mental health services.

Descriptives and semi-structured interview. Basic descriptive information was collected from the young person's social worker, as part of the larger quantitative study. This included the age and reason that the young people had entered care. The semi-structured interview was developed based on the literature on qualitative interviewing techniques and qualitative and quantitative work in the child mental health field [e.g., 9, 21-22]. Interviews broadly covered four core areas: (i) views on what is broadly helpful and unhelpful within the care system, (ii) coping and support for children's mental health and wellbeing (including at home, within social-care, and experiences around mental health services), (iii) coping and

For peer review only - http://bmjopen.bmj.com/site/about/guidelines.xhtml

support for trauma-related mental health more specifically (e.g., how young people cope with memories of their maltreatment), and (iv) views on ideal support. Questions were primarily focused on the young person's subjective experiences (see Appendix 1 for interview schedule). Additionally, participants were also asked what advice they might give a friend in a similar situation, to elicit broader information from young people who might have struggled to answer these questions about themselves. Questions were primarily open-ended (e.g., "If you're having a hard time what do you do to make yourself feel better or different?"). Some direct prompting and questioning were used to clarify comments and gain further insight (e.g., "Was that helpful?"). Interviews lasted from 6-32 minutes (M = 15.36 minutes).

Data Analyses

Transcripts were analysed using a reflexive thematic analysis approach [23-24] in NVivo 12. Given the exploratory nature of the study, thematic analysis was chosen as the data analytic approach as it allows for a detailed exploration of the data and does not rely on a specific theoretical framework. Co-author (ER), who was not involved in any of the interviews or the broader quantitative study, coded all transcripts. While the purpose of qualitative research is not to completely remove or minimise the influence of researcher subjectivity, a coder who was had not been involved in study design or data collection was used, to reduce potential biases. She first read all transcripts to gain an understanding of the overall data (data immersion) and then systematically coded each transcript using an iterative process [24]. Following the systematic coding of all transcripts, codes were grouped to form overarching themes. The broader themes were also separated into subthemes, to accurately capture more fine grain information provided by participants and ensure the themes appropriately reflected different viewpoints. Co-author (TH) independently explored all (un-coded) transcripts and generated their own key themes. A consensus meeting showed strong agreement between the primary themes. There was not capacity within the study to seek

For peer review only - http://bmjopen.bmj.com/site/about/guidelines.xhtml

For peer review only

further input from participants at this point, however reflective practices were used throughout all interviews to ensure clear understanding of the discussion and to minimise any chance of misinterpreting what was being said (e.g., reflecting, summarising back to participants and seeking further clarification where needed).

Patient and Public Involvement

This study occurred as part of a series of projects, which involves extensive consultation with services, as well as feedback from care-experienced young people and foster carers. This feedback particularly shaped the research questions and use of qualitative methods. Young people and carers were not involved in recruitment for the project. There has been wide written and verbal feedback to stakeholders as part of the larger project.

Results

Descriptive Information

The sample comprised of 14 females and 11 males, aged 10 to 16 years old. They had entered care between infancy and 16 years old, and had been in care from 6 months to almost 14 years, while 80% of the sample had had more than one placement since entering care. Most (76%) were living in a foster placement with a non-biological carer, while four were in a kinship placement (e.g., with a grandmother), and two lived in residential care homes. All were in care due to the experience of abuse and/or neglect.

Interview Themes

Thematic analysis identified three primary themes covering the broader experience of being in care (Theme 1); the centrality of social support to wellbeing and mixed views on professional help (Theme 2); and the use of both adaptive and maladaptive day-to-day coping strategies (Theme 3). A list of themes and subthemes can be found in Table 2.

Theme 1: Being in care could create a sense of safety but some young people struggled with a lack of control and agency

For peer review only - http://bmjopen.bmj.com/site/about/guidelines.xhtml

When asked what it was like to be in care, most, but not all participants said that being in care was a better or safer alternative to living with their biological family and that they felt safer in care. However, almost all discussed occasions of feeling stigmatised, as well as experiences of being poorly supported by services.

Subtheme 1: Positives to being in care included a sense of safety and being part of a family. The majority of participants reported that feeling safe and secure was a particularly positive aspect of being in care. Some participants also described experiencing a sense of relief when first coming into care, recognising that it was not appropriate for them to remain in an unsafe environment.

Relieved actually from going to a bad environment into somewhere I thought could be quite safe. [F; 16yo]

To know at the end of the day you can go home, instead of thinking 'OK, I'm going to go home tonight, am I going to find my mum crying on the floor again drunk?' [F; 12yo]

The majority of young people discussed how they have felt "normal" and like part of a family since coming into care. This was expressed regardless of participants' current relationships with biological family, with many describing strong and important bonds with both their biological and foster family. Feeling like part of a family was especially valued and some expressed their appreciation for the support and sense of belonging provided by their foster carers.

Just feel like an ordinary family really, like an ordinary person. Sometimes I don't even realise I'm in care. [11yo]

I'm lucky I ended up here. I'm so goddamn lucky I ended up here, coz this is my home now, it's my family and I love 'em, I love 'em, I actually do, coz they're my family. [12yo]

For peer review only - http://bmjopen.bmj.com/site/about/guidelines.xhtml

In some cases, these positive aspects of their placement were reported to facilitate improvements within different areas of participants' lives, such as their school, and their ability to cope with difficult situations and emotions.

They [carers] *like understand what I'm saying and like if I'm annoyed they help me to know how to calm down.* [10yo]

I started knuckling down at school, because I felt safe. [12yo]

Subtheme 2: Negative aspects of being in care included feeling different and not being listened to or respected. Most participants discussed feeling "different" because they were a young person in care, and were concerned about others' judgement. Mandatory forms and compulsory meetings with social workers could create difficult situations for participants, or *"annoying"* disruptions, which served as regular reminders that they were different from other children, who were not asked to fill in forms and (for example) leave class to meet with a social worker. Alongside this, concerns about others' (e.g., teachers, peers) lack of understanding meant that many participants were very conscious of being judged or stigmatised, particularly within the school context.

Most people think you are naughty 'cause you're in care. [10yo]

Like the social workers coming in here [school] or whatever… it's awkward 'cause like a normal person wouldn't really have that. [10yo]

Amongst adolescents and particularly those struggling with mental health difficulties, feeling different from their peers was often accompanied by complaints of lacking freedom and control, generating a feeling of restricted independence. This led some older participants to express their desire to leave the care system and move into independent living, in order to take back control over their lives.

I have no control over my own life. [16yo]

For peer review only - http://bmjopen.bmj.com/site/about/guidelines.xhtml

I want to go into independent living, because I've had enough of living with, like, parent figures and I just want to be on my own and be independent and prove that I can do it. [16yo]

Theme 2: Social support was considered key to coping but not always available, while access to professional support was inconsistent. The majority of participants most valued informal day-to-day support from a trusted adult, while describing formal/professional support to be less available and less helpful. Many participants expressed the need for professional support to be improved and made more readily available.

Subtheme 1: Day-to-day support from a trusted adult was important for wellbeing. Having a trusted adult to talk to about day-to-day problems or concerns, whenever they felt they needed to, was identified as the most valuable form of support for participants. Particular aspects valued in a trusted adult were their ability to listen, to *"keep secrets"* and to *"give good advice".* The importance of being able to call upon this support as and when it was needed was often emphasised. For example, participants described the benefits of being able to talk to carers after a bad day or to speak with key staff at school if there was an argument in a lesson. Knowing there was always a trusted adult available to speak to was central to maintaining wellbeing for many participants.

Telling [your worries/concerns to] *someone you trust makes you a lot calmer*. [F; 16yo]

Overall, carers were by far reported as the primary source of support from the perspective of most of the participants. Some participants also talked about their social worker or a school staff member being key sources of support. For older teens, some also mentioned close friends as their key source of support, particularly if they viewed friends as more trustworthy than adults.

..most foster carers are very nice and they really help you with your life… they look after and like you. [10yo]

For peer review only - http://bmjopen.bmj.com/site/about/guidelines.xhtml

> *Whenever something* [is] *tough, I'll tell her* [social worker] *and she'd always take me out and then we'd always chat about something different and then she'd take my mind off it.* [16yo]

> *My school nurse helped me a lot because whenever I had a problem I'd go to her and she'd try to sort it out as quick as possible.* [16yo]

> *Really close friends, I can trust them with my life.* [15yo]

While there were some cases of very positive relationships between a young person and their social worker, perceived ineffective communication between social workers and young people was identified as one of their central problems in terms of feeling supported. A focus of many interviews was how transient their social worker was, with almost all having multiple different social workers over their time in care, or even over the past year. This transience made it particularly difficult for young people to build trust, which could also influence their willingness to disclose information about their early experiences and their general trust of professionals. Some also described situations where they believed that their confidentiality had been breached by adults in their support network, further resulting in them feeling let down and their trust being eroded.

> *I just want one […] one person [Social Worker] who is going to be there for quite a long time and not going to leave all of a sudden and I can build up a bond.* [15yo]

> *You tell them [about your experiences] and then they leave and you can't tell them anymore. So you're like oh my God who can I tell? Who can I trust? It's hard.* [16yo]

A minority of participants perceived that there was no one they could talk to. These participants were all adolescents and all reported struggling with significant mental health difficulties, including engaging in self-harm and/or drug or alcohol use. For these young people, the lack of a trusted adult, which largely included an unstable placement, was particularly problematic. Perceived unhelpful foster placements were discussed in terms of

For peer review only - http://bmjopen.bmj.com/site/about/guidelines.xhtml

young people perceiving carers fostering *"for the money",* blaming or not trusting the young person, not putting effort in to spending time with them, or the young person perceiving that the carer had not been appropriately trained.

> *Since I was seven all I've ever done is either sat on the sofa and watched tv on my own coz no one talks to me or I've sat in my room and watched tv on my own because nobody ever talks to me* [16yo]

Subtheme 2: Formal mental health support was often unavailable and opinions on usefulness were mixed. Formal support from mental health professionals was identified as being often unavailable and unhelpful. For those who had seen a mental health professional, this was often the school counsellor, with the minority accessing formal child and adolescent mental health services (CAMHS). Among those who had received support, either in the form of a school counsellor or CAMHS, opinions were mixed, with a slight majority describing this support as unhelpful. Young people had difficultly specifically identifying what they found unhelpful about this support, although some did describe feeling restricted to a scheduled appointment and having to talk about their past despite not wanting to do so.

> *It made it worse like, having a scheduled time to go and talk about things when I don't really want to talk about them, like I'd rather just say it when I felt like saying it. It just made me think about it more which made it worse.* [14yo]

Similarly, those who had positive experiences with mental health professionals struggled to identify what it was that they had done that had been helpful, although some of them did highlight that practitioners had provided them with a safe space to talk and help them cope.

> *It made me talk to people and like actually not just keep everything to yourself.* [15yo]
>
> *I got my feelings clear to everyone.* [11yo]

Theme 3: Talking about problems was seen as important, but maladaptive coping strategies were also reported.

For peer review only - http://bmjopen.bmj.com/site/about/guidelines.xhtml

When participants were asked what they did when experiencing negative emotions, a range of coping strategies were identified. Some of these strategies were adaptive, such as talking to somebody they trusted, and some were maladaptive, such as self-harm.

Subtheme 1: Talking about worries or challenges was identified as a helpful strategy for coping with negative emotions. Talking about day-to-day worries or problems with a trusted person was identified as a common strategy used in response to negative emotions, such as sadness, anger, or anxiety. As previously highlighted, carers, teachers and social workers were amongst those who participants felt comfortable speaking to. This was discussed as a primarily helpful strategy, often resulting in feeling better afterwards or enabling problems to be resolved.

If you're feeling down, always talk to someone that you're close to about it, never hold it in. [16yo]

Subtheme 2: A majority avoided talking about their pre-care experiences and in the context of mental health difficulties maladaptive coping was common. While talking to someone about current worries was seen as important for coping, most participants thought it was best to avoid talking about their pre-care experiences. Indeed, when asked what advice they might give a friend who was struggling, many endorsed they would encourage them to talk to someone, but then also endorsed that this was not something they themselves did.

Speak up and tell people how you are feeling and what you've been through, and that you're struggling to cope. Because if people don't know people can't help.

Interviewer: Did you do that?

No, because I didn't know them and I don't trust people I don't know. [16yo]

Reasons for avoiding discussing their maltreatment varied but the most common justifications were either because talking about pre-care experiences made them feel uncomfortable or because they did not think it would be helpful. This sense of discomfort

For peer review only

when talking about previous experiences often appeared to be associated with a lack of trust in adults and services. Participants also discussed feeling embarrassed, uncomfortable with the amount of attention placed on them, and unsure of how to act when describing events.

I don't really like talking about my past too much, it's, it's like all attentions on me and it's like awkward. [14yo]

It don't change nothing, it don't mean it go back. It still happened you can't make it [go away]. [10yo]

From those who identified talking as an unhelpful strategy, many held the attitude that, as events were in the past, talking about them would not change anything. Some also felt that such discussion acted as a barrier to moving forward. This was particularly reported by young people who were experiencing serious mental health difficulties, including current distressing intrusive memories of their maltreatment.

I'm not saying "suck it up and be a man" but just try and just keep your mind off it, keep doing what you're doing cos you don't really want to let the past like take over you. Because the past is in the past and I really don't want to bring my past up. I'm trying to move forward for the minute but obviously if people are bringing it up, I can't. [15yo]

In general, many participants endorsed trying not to think about their negative (e.g., maltreatment) memories and avoidance of talking about their past as a primary way to cope. In most cases this involved distracting themselves with activities, such as watching TV and spending time with friends. Indeed in many cases when talking about their support systems, young people endorsed that adults helping to distract them was helpful (e.g changing the topic; social workers taking them to lunch; see Theme 2 quotes). These distractions shifted the focus of their attention onto something else, allowing them to avoid thinking about their early experiences.

[in response to question about coping] *just watch tv or something.* [10yo]

For peer review only - http://bmjopen.bmj.com/site/about/guidelines.xhtml

I would go on the PlayStation and just kill people. [15yo]

In the case of adolescents who were struggling with mental health difficulties, there were more frequent examples of destructive coping strategies, particularly self-harm and drug and alcohol use. Many of these young people reported that they did not have any other way of coping with their emotional difficulties.

I pretend to be someone I'm not. I pretend to be okay when I'm not and let emotions build up until eventually I just break. [12yo]

I cry and I hurt myself that's literally the only way I know how to deal with things anymore. [16yo]

For peer review only

Discussion

This study aimed to learn more about the views of young people in out-of-home care, in relation to coping, support, and help-seeking with emotional difficulties. Across the interviews there were core common themes, including the importance of having a consistent and trusted adult with whom they could share their worries. Carers were identified as the primary source of emotional support by most young people. While many participants talked about the importance of talking to your support system as a means of coping, most also thought that it was not helpful to talk about their pre-care experiences. Adolescents experiencing mental health difficulties also commonly struggled to identify adaptive coping strategies or support systems.

Similar to other qualitative work with young people in care [9,12], participants viewed the support of a trusted adult as key to coping with daily stress and interpersonal difficulties, and with more pervasive mental health difficulties. In the vast majority of cases, the carer (or a key worker if in residential care) was viewed as the primary source of this support, although some young people talked about this support also coming from their social worker, a trusted staff member at school, or friend. Trustworthiness was discussed as

For peer review only - http://bmjopen.bmj.com/site/about/guidelines.xhtml

particularly central to the young person feeling supported, including the perception that the person would be able to keep information confidential. A lack of trust was a key concern and potential barrier to seeking help. While some young people had positive relationships with their social workers, many participants discussed changes in social workers as another key barrier to building a trusted relationship and seeking support for emotional difficulties. This reflects similar findings by Wood and Selwyn in their qualitative work, where young people in care also discussed the challenges of navigating regular changes in social workers and the ultimate perceived negative impact on their subjective wellbeing [25]. Findings highlight the need for services to explore policies around mitigating some of the consequences of social worker changes on the young person's wellbeing. Social work has some of the highest burn-out and turn-over rates of any profession. Providing more robust training and support to social workers is considered key to reducing social work changes [26]. However, given some change is inevitable, policies for how these changes happen are crucial, to minimise the potential eroding of trust. Given the age range included adolescents, it was interesting that peers were less commonly identified as a primary source of support. In adolescence, research has shown that friendships can be central to resilient functioning [27]. The lack of focus on peers may be due to the concern young people in care expressed about being stigmatised by peers for being in care, meaning they are less likely to turn to friends. It may also be that some, especially teens experiencing mental health difficulties, struggled in school in general and had changed schools during their time in care. The role of friendship in promoting resilience in this group of young people is worthy of further exploration.

While many participants talked about going to their carer for support if they were feeling worried or upset (e.g., if they were upset about something at school), many at the same time emphatically stated that they did not think it was useful to talk about their maltreatment experiences, with either their carer or mental health professionals. While this

For peer review only - http://bmjopen.bmj.com/site/about/guidelines.xhtml

was the case across much of the sample, it is perhaps most challenging for those young people who discussed experiencing significant mental health difficulties. This sub-set of the sample most commonly reported feeling that they had no person they could turn to for support, that they did not want to talk to a professional about their maltreatment or mental health, and had no strategies for coping with their mental health beyond self-harm and drug and alcohol abuse. Beliefs around not talking about maltreatment or trauma present a particular challenge to mental health and social-care professionals. Of course, young people could and should not be forced to talk about traumatic experiences. Some may also be coping fine and not need to talk through their experiences. It is also completely understandable that young people may not want to talk about distressing early experiences. Yet, avoiding talking or thinking about traumatic experiences (i.e., thought suppression) is a key maintainer of poor mental health outcomes [15], including for young people in care [16]. Further, a key component of evidenced-based trauma-focused psychological support involves processing the traumatic experiences, commonly via a trauma narrative [e.g., 28]. There remains an urgent need to understand how services can best engage young people in care who may benefit from professional support in treatments that might involve talking about their experiences, particularly for a group where developing trust and a strong therapeutic relationship is essential [10]. This may be partly achieved by having systems, including at a social-care and education level, where staff are appropriately trained and supported in engaging with these challenging conversations with young people from earlier on in their time in care, so young people know that it is safe to talk about these experiences if they want to. Many services in the UK would report using "trauma-informed" approaches to their practice [29], which would include creating safe spaces for young people to talk about their experiences. However, the evidence-base for how these approaches are genuinely implemented and their effectiveness, remains scarce. Relatedly, many young people reported feeling stigmatised by being in care,

For peer review only - http://bmjopen.bmj.com/site/about/guidelines.xhtml

including by professionals (e.g., teachers) and peers – a finding that is also in line with other qualitative work with this group [e.g., 26]. It will therefore also be important for services to consider how to address issues of stigma, which can be a barrier to help-seeking. Strength and relationship-based approaches may be useful here [30], but remain poorly researched in terms of how they are used in practice or their general effectiveness. These remain important areas for future research, to focus not just on targeting psychopathology but more broadly improving the emotional wellbeing of these young people.

For the minority of participants in this study who had received professional mental health support, including via CAMHS and school counselling, views on this support were mixed. In their 2008 review of qualitative literature on children in care and mental health services, Davies and Wright highlighted that many young people felt wary and unsure of professional mental health support, and they highlighted the importance of developing trust [9]. Ten years later, the current study found that young people in care are still experiencing these same concerns. There remains an urgent need to improve understanding of the service-level barriers to accessing professional support, including access to evidence-based psychological interventions (e.g., see review [31]). Within the current UK CAMHS system, thresholds for professional mental health support often mean young people are in crisis, with complex mental health needs, by the time they can access support (e.g., see [32]). Exploring feasible but appropriate avenues for providing effective evidence-based support, before reaching crisis, is clearly an important avenue for addressing the mental health needs of these young people.

Limitations

While this study has key strengths, including a focus on an under-researched group, and the use of qualitative interviews to provide an in-depth exploration of their views on coping and wellbeing, findings should also be considered in light of key limitations. First, we

For peer review only - http://bmjopen.bmj.com/site/about/guidelines.xhtml

used opportunity sampling methods, which potentially creates sampling bias. All young people were participants in a larger quantitative study of mental health and wellbeing [see ref 16]. Young people who did not want to talk about their experiences and mental health would likely have declined participating in this study. Participants also all came from two local authorities in England. While findings cannot necessarily be generalised to other young people in out-of-home care, it is worth noting the significant overlap between these findings and qualitative work with young people in care and foster carers in the UK, US, and Australia [e.g., 9,19]. Finally, quantitative research is needed to provide more robust evidence on the consequences of different support systems and coping strategies in relation to the mental health of these young people.

Conclusions

In sum, while there were positives to being in care and many young people felt well supported by their carer, results of this qualitative study identified key areas to be addressed to improve support for the emotional wellbeing of this group. This included improving the continuity of social workers and carers to allow young people to develop trusted relationships with key adults, targeting broader concerns around stigma and trust (including in the school system) that may act as a barrier to help-seeking, and ensuring young people are receiving appropriate evidence-based psychological support in a more timely manner, before reaching crisis point. Addressing these issues remains a significant challenge for services, particularly alongside long-term underfunding of mental health services. Yet, failing to provide adequate support, both in terms of informal day-to-day support and professional services, can have a substantial impact on the trajectory of these young people.

For peer review only - http://bmjopen.bmj.com/site/about/guidelines.xhtml

Contributors: RMH is the lead researcher on the project and PI on the ESRC grant. She led the conceptualisation of the project, conducted a proportion of the interviews, and was responsible for overall project management. SLH was involved in the conceptualisation of the project, including the design of the interview schedule and also contributed to discussion on the themes and feedback on the manuscript. RM-S was involved in the conceptualisation of the project and contributed to the discussion about the themes and feedback on the manuscript. EE was the research assistant on the project and conducted a proportion of the interviews and contributed to transcribing, data analysis, and feedback on the manuscript. ER-E was involved in the transcribing, contributed to data analysis and provided feedback on the manuscript. TH contributed to transcribing and feedback on the codes and themes.

The authors also wish to sincerely thank the many social-care and mental health service staff, carers, and young people who contributed to the design of this project and from whom we continue to learn so much.

Funding: This research was funded by an ESRC Future Leader Grant (ES/N01782X/1) and University of Bath Prize Fellowship awarded to RMH.

Competing Interests: None to declare.

Data sharing: Anonymised transcripts will be available on request on a case-by-case basis.

For peer review only - http://bmjopen.bmj.com/site/about/guidelines.xhtml

References

[1] Department for Education. Statistics: looked after children. London: Author. 2018.

[2] Pecora PJ, White CR, Jackson LJ, Wiggins T. Mental health of current and former recipients of foster care: A review of recent studies in the USA. Child & Family Social Work. 2009; 14(2):132-146.

[3] Hiller RM, St Clair MCS. The emotional and behavioural symptom trajectories of children in long-term out-of-home care in an English local authority. Child Abuse & Neglect. 2018; 81:106-117.

[4] Newton RR, Litrownik AJ, Landsverk JA. Children and youth in foster care: Disentangling the relationship between problem behaviors and number of placements. Child Abuse & Neglect. 2000; 24(10):1363-1374.

[5] Ford T, Vostanis P, Meltzer H, Goodman R. Psychiatric disorder among British children looked after by local authorities: comparison with children living in private households. The British Journal of Psychiatry. 2007; 190(4):319-325.

[6] McMillen JC, Zima BT, Scott Jr LD, Auslander WF, Munson MR, Tollie MT, Spitznagel EL. Prevalence of psychiatric disorders among older youths in the foster care system. Journal of the American Academy of Child & Adolescent Psychiatry. 2005; 44(1):88-95.

[7] Oswald SH, Heil K, Goldbeck L. History of maltreatment and mental health problems in foster children: A review of the literature. Journal of Pediatric Psychology. 2009; 35(5):462-472.

[8] Jones R, Everson-Hock ES, Papaioannou D, Guillaume L, Goyder E, Chilcott J, ... & Swann C. Factors associated with outcomes for looked-after children and young people: a correlates review of the literature. Child: Care, Health and Development. 2011; 37(5):613-622.

For peer review only - http://bmjopen.bmj.com/site/about/guidelines.xhtml

[9] Davies J, Wright J. Children's voices: A review of the literature pertinent to looked-after children's views of mental health services. Child and Adolescent Mental Health. 2008; 13(1):26-31.

[10] Shaw M, De Jong M. Child abuse and neglect: a major public health issue and the role of child and adolescent mental health services. The Psychiatrist. 2012; 36(9):321-325.

[11] Ahmed K, Windsor L, Scott S. In their own words: abused children's perceptions of care provided by their birth parents and foster carers. Adoption & Fostering. 2015; 39(1): 21-37.

[12] Sinclair I, Wilson K. Matches and mismatches: The contribution of carers and children to the success of foster placements. British Journal of Social Work. 2003; 33(7):871-884.

[13] Rock S, Michelson D, Thomson S, Day C. Understanding foster placement instability for looked after children: A systematic review and narrative synthesis of quantitative and qualitative evidence. British Journal of Social Work. 2013; 45(1):177-203.

[14] Murray L, Creswell C, Cooper PJ. The development of anxiety disorders in childhood: an integrative review. Psychological Medicine. 2009; 39(9):1413-1423.

[15] Trickey D, Siddaway AP, Meiser-Stedman R, Serpell L, Field AP. A meta-analysis of risk factors for post-traumatic stress disorder in children and adolescents. Clinical Psychology Review. 2012; 32(2):122-138.

[16] Hiller RM, Meiser-Stedman R, Elliott E, Banting R, Halligan SL. Cognitive predictors of (complex) posttraumatic stress: A longitudinal study of young people in out-of-home care. Journal of Child Psychology and Psychiatry. 2020; In Press.

[17] Marshall B, Cardon P, Poddar A, Fontenot R. Does sample size matter in qualitative research?: A review of qualitative interviews in IS research. Journal of computer information systems. 2013; 54(1):11-22.

[18] Vasileiou K, Barnett J, Thorpe S, Young T. Characterising and justifying

For peer review only - http://bmjopen.bmj.com/site/about/guidelines.xhtml

sample size sufficiency in interview-based studies: systematic analysis of qualitative health research over a 15-year period. BMC Medical Research Methodology. 2018; 18(1):148.

[19] Ahrens KR, DuBois DL, Garrison M, Spencer R, Richardson LP, Lozano P. Qualitative exploration of relationships with important non-parental adults in the lives of youth in foster care. Children and Youth Services Review. 2011; 33(6):1012-1023.

[20] Johnson E, Menna R. Help seeking among adolescents in foster care: A qualitative study. Children and Youth Services Review. 2017; 76:92-99.

[21] Docherty S, Sandelowski M. Focus on qualitative methods: Interviewing children. Research in Nursing & Health. 1999; 22(2):177-185.

[22] Van Wesel F, Boeije H, Alisic E, Drost S. I'll be working my way back: A qualitative synthesis on the trauma experience of children. Psychological Trauma: Theory, Research, Practice, and Policy. 2012; 4(5):516.

[23] Braun V, Clarke V. Using thematic analysis in psychology. Qualitative Research in Psychology. 2006; 3(2):77-101.

[24] Braun V, Clarke V, Hayfield N, Terry G. Thematic analysis. In P Liamputtong. Handbook of Research Methods in Health Social Sciences. Singapore: Springer. 2019. 843-60.

[25] Wood M, Selwyn J. Looked after children and young people's views on what matters to their subjective well-being. Adoption & Fostering. 2017; 41(1):20-34.

[26] Kim H, Ji J, Kao D. Burnout and physical health among social workers: A three-year longitudinal study. Social Work. 2011; 56(3):258-268.

[27] Van Harmelen AL, Kievit RA, Ioannidis K, Neufeld S, Jones PB, Bullmore E, Dolan R, Fonagy P, Goodyer I, NSPN Consortium. Adolescent friendships predict later resilient functioning across psychosocial domains in a healthy community cohort. Psychological Medicine. 2017;47(13):2312-22.

For peer review only - http://bmjopen.bmj.com/site/about/guidelines.xhtml

[28] Deblinger E, Mannarino AP, Cohen JA, Runyon MK, Steer RA. Trauma-focused cognitive behavioral therapy for children: impact of the trauma narrative and treatment length. Depression and Anxiety. 2011; 28(1):67-75.

[29] Beyerlein BA, Bloch E. Need for trauma-informed care within the foster care system: A policy issue. Child Welfare. 2014; 93(3):7.

[30] Brooks R, Brooks S. Creating resilient mindsets in children and adolescents: A strength-based approach for clinical and nonclinical populations. In Resilience interventions for youth in diverse populations. Springer, New York, NY. 2014; 59-82.

[31] Leve LD, Harold GT, Chamberlain P, Landsverk JA, Fisher PA, Vostanis P. Practitioner review: children in foster care–vulnerabilities and evidence-based interventions that promote resilience processes. Journal of Child Psychology and Psychiatry. 2012; 53(12): 1197-1211.

[32] Blow E. Children and young people's mental health – policy, CAMHS services, funding and education. London: House of Commons Library. 2015.

For peer review only

For peer review only - http://bmjopen.bmj.com/site/about/guidelines.xhtml

Table 1. *Sample demographics.*

**Young person descriptives (N = 25)**	
Age, *M* (*SD*), Range	13.0 (2.24)
Sex (% female)	56%
Ethnicity (% Caucasian)	80%
Age entered care, *M* (*SD*)	8.7 yrs (4.2)
Time in care, *M* (*SD*)	4.2 yrs (3.6)
% who had multiple placements	80%
Type of placement	
Non-biological foster carer	76%
Biological foster carer (e.g., grandmother)	16%
Residential care home	8%

For peer review only

For peer review only - http://bmjopen.bmj.com/site/about/guidelines.xhtml

Table 2. *Themes and subthemes from thematic analysis.*

Themes	Sub-themes
**Being in care could create a sense of safety but some young people struggled with a lack of control and agency**	Positive aspects of being in care were the sense of safety and being part of a family.
	Negative aspects of being in care included feeling different and not being listened to or respected.
**Social support was considered key to coping but not always available, while access to professional support was inconsistent.**	Day-to-day support from a trusted adult was identified as very important for wellbeing.
	Formal mental health support was often unavailable and opinions on usefulness were mixed.
**Talking about problems was seen as important, but maladaptive coping strategies were also reported**	Talking about worries or challenges was identified as a helpful strategy for coping with negative emotions.
	A majority avoided talking about their pre-care experiences and in the context of mental health difficulties maladaptive coping was common.

For peer review only

For peer review only - http://bmjopen.bmj.com/site/about/guidelines.xhtml

Child qualitative interview

Thank you for talking to me today. Like it said in the letter I'm interested in finding out a bit more about your views on how you cope and where you might go for support or help if you're struggling with thoughts or feelings. Remember, you don't need to talk about your experiences in your home. You can if you want to but we won't be asking you to. What I'm really interested in is what happened *after* this, when you came in to care. This isn't like a test at school, there are no right or wrong answers, it's just about talking honestly and sharing your opinion. If you want to stop at any time or take a break just let me know. Does that sound ok?

Demographics

- Do you remember how old you were when you first came in to care?
- How long have you lived in [current placement]?

Reactions

- How have you felt since you came in to care?
    - Are there good parts?
    - What are some of the harder parts?

Support

- If you're having a hard time, is there anything you do to try and make yourself feel better or different?
    - (*example might be argument with friend at school; through to mental health difficulty if yp has disclosed this*)

- Is there anyone you talk to, to try to make yourself feel better?
    - Who? Is that helpful/unhelpful? Why?
    - Anyone else?
    - Have you ever talked to a professional? Like a psychologist or school counsellor? What was this like? What was helpful/unhelpful?

- Do you ever talk to anyone about what you experienced before you came into care?
    - Why/why not?
    - What helps/doesn't help?

Support wishes

- What support do you wish you had got that you didn't?
    - (example might be – *if you were asked to create the best possible care system to support young people who were struggling with their thoughts and feelings, what would it look like?*)
- If you had a friend that went through the same things as you, how would you help them? What advice might you give them to help them cope with their thoughts and feelings?

Is there anything else you want to add that we haven't covered?

For peer review only - http://bmjopen.bmj.com/site/about/guidelines.xhtml

Reporting checklist for qualitative study.

Based on the SRQR guidelines.

Instructions to authors

Complete this checklist by entering the page numbers from your manuscript where readers will find each of the items listed below.

Your article may not currently address all the items on the checklist. Please modify your text to include the missing information. If you are certain that an item does not apply, please write "n/a" and provide a short explanation.

Upload your completed checklist as an extra file when you submit to a journal.

In your methods section, say that you used the SRQRreporting guidelines, and cite them as:

O'Brien BC, Harris IB, Beckman TJ, Reed DA, Cook DA. Standards for reporting qualitative research: a synthesis of recommendations. Acad Med. 2014;89(9):1245-1251.

		Reporting Item	Page Number
**Title**			
	#1	Concise description of the nature and topic of the study identifying the study as qualitative or indicating the approach (e.g. ethnography, grounded theory) or data collection methods (e.g. interview, focus group) is recommended	1

For peer review only

**Abstract**			
	#2	Summary of the key elements of the study using the abstract format of the intended publication; typically includes background, purpose, methods, results and conclusions	2
**Introduction**			
Problem formulation	#3	Description and signifcance of the problem / phenomenon studied: review of relevant theory and empirical work; problem statement	4-5
Purpose or research question	#4	Purpose of the study and specific objectives or questions	5
**Methods**			
Qualitative approach and research paradigm	#5	Qualitative approach (e.g. ethnography, grounded theory, case study, phenomenolgy, narrative research) and guiding theory if appropriate; identifying the research paradigm (e.g. postpositivist, constructivist / interpretivist) is also recommended; rationale. The rationale should briefly discuss the justification for choosing that theory, approach, method or technique rather than other options available; the assumptions and limitations implicit in those choices and how those choices influence study conclusions and transferability.	7-8

For peer review only

		As appropriate the rationale for several items might be discussed together.	
Researcher characteristics and reflexivity	#6	Researchers' characteristics that may influence the research, including personal attributes, qualifications / experience, relationship with participants, assumptions and / or presuppositions; potential or actual interaction between researchers' characteristics and the research questions, approach, methods, results and / or transferability	7
Context	#7	Setting / site and salient contextual factors; rationale	6
Sampling strategy	#8	How and why research participants, documents, or events were selected; criteria for deciding when no further sampling was necessary (e.g. sampling saturation); rationale	6
Ethical issues pertaining to human subjects	#9	Documentation of approval by an appropriate ethics review board and participant consent, or explanation for lack thereof; other confidentiality and data security issues	6
Data collection methods	#10	Types of data collected; details of data collection procedures including (as appropriate) start and stop dates of data collection and analysis, iterative process, triangulation of sources / methods, and modification of procedures in response to evolving study findings; rationale	6

For peer review only

Data collection instruments and technologies	#11	Description of instruments (e.g. interview guides, questionnaires) and devices (e.g. audio recorders) used for data collection; if / how the instruments(s) changed over the course of the study	6-8
Units of study	#12	Number and relevant characteristics of participants, documents, or events included in the study; level of participation (could be reported in results)	6-8
Data processing	#13	Methods for processing data prior to and during analysis, including transcription, data entry, data management and security, verification of data integrity, data coding, and anonymisation / deidentification of excerpts	6-8
Data analysis	#14	Process by which inferences, themes, etc. were identified and developed, including the researchers involved in data analysis; usually references a specific paradigm or approach; rationale	6-8
Techniques to enhance trustworthiness	#15	Techniques to enhance trustworthiness and credibility of data analysis (e.g. member checking, audit trail, triangulation); rationale	7-8
**Results/findings**			
Syntheses and interpretation	#16	Main findings (e.g. interpretations, inferences, and themes); might include development of a theory or model, or integration with prior research or theory	8-16

For peer review only

Links to empirical data	#17	Evidence (e.g. quotes, field notes, text excerpts, photographs) to substantiate analytic findings	8-16
**Discussion**			
Intergration with prior work, implications, transferability and contribution(s) to the field	#18	Short summary of main findings; explanation of how findings and conclusions connect to, support, elaborate on, or challenge conclusions of earlier scholarship; discussion of scope of application / generalizability; identification of unique contributions(s) to scholarship in a discipline or field	16
Limitations	#19	Trustworthiness and limitations of findings	19
**Other**			
Conflicts of interest	#20	Potential sources of influence of perceived influence on study conduct and conclusions; how these were managed	21
Funding	#21	Sources of funding and other support; role of funders in data collection, interpretation and reporting	21

Notes:

- 21: n/a (blinded) The SRQR checklist is distributed with permission of Wolters Kluwer © 2014 by the Association of American Medical Colleges. This checklist was completed on 30. July 2019 using https://www.goodreports.org/, a tool made by the EQUATOR Network in collaboration with Penelope.ai

For peer review only
